# Imaging of heart disease in women: review and case presentation

**DOI:** 10.1007/s00259-022-05914-6

**Published:** 2022-08-17

**Authors:** Nidaa Mikail, Alexia Rossi, Susan Bengs, Achi Haider, Barbara E. Stähli, Angela Portmann, Alessio Imperiale, Valerie Treyer, Alexander Meisel, Aju P. Pazhenkottil, Michael Messerli, Vera Regitz-Zagrosek, Philipp A. Kaufmann, Ronny R. Buechel, Cathérine Gebhard

**Affiliations:** 1 Department of Nuclear Medicine, University Hospital Zurich, Raemistrasse 100, 8091 Zurich, Switzerland; 2 Center for Molecular Cardiology, University of Zurich, Schlieren, Switzerland; 3 Division of Nuclear Medicine and Molecular Imaging, Department of Radiology, Massachusetts General Hospital, Harvard Medical School, Boston, MA USA; 4 Department of Cardiology, University Heart Center, University Hospital Zurich, Zurich, Switzerland; 5 Nuclear Medicine and Molecular Imaging - Institut de Cancérologie de Strasbourg Europe (ICANS), University of Strasbourg, Strasbourg, France; 6 Molecular Imaging – DRHIM, IPHC, UMR 7178, CNRS/Unistra, Strasbourg, France; 7 Charité, Universitätsmedizin, Berlin, Berlin Germany; 8 University of Zurich, Zurich, Switzerland; 9 Division of Cardiology, Department of Internal Medicine II, Medical University of Vienna, Vienna, Austria

**Keywords:** Noninvasive imaging, SPECT, PET, CMR, CCTA, Sex, Gender

## Abstract

Cardiovascular diseases (CVD) remain the leading cause of mortality worldwide. Although major diagnostic and therapeutic advances have significantly improved the prognosis of patients with CVD in the past decades, these advances have less benefited women than age-matched men. Noninvasive cardiac imaging plays a key role in the diagnosis of CVD. Despite shared imaging features and strategies between both sexes, there are critical sex disparities that warrant careful consideration, related to the selection of the most suited imaging techniques, to technical limitations, and to specific diseases that are overrepresented in the female population. Taking these sex disparities into consideration holds promise to improve management and alleviate the burden of CVD in women. In this review, we summarize the specific features of cardiac imaging in four of the most common presentations of CVD in the female population including coronary artery disease, heart failure, pregnancy complications, and heart disease in oncology, thereby highlighting contemporary strengths and limitations. We further propose diagnostic algorithms tailored to women that might help in selecting the most appropriate imaging modality.

## Introduction

Noninvasive imaging is of paramount value for the diagnosis and management of cardiovascular diseases (CVD). Indeed, there is a wealth of evidence showing that the appropriate choice of imaging modality improves not only diagnostic accuracy but also long-term outcomes [1]. Although overall diagnostic strategies are comparable between sexes, female-specific attributes may substantially affect the diagnostic performance of the underlying procedure (Table 1). Furthermore, technical challenges due to breast attenuation and general radiation safety considerations constitute major decision-making criteria for the selection of the most appropriate diagnostic procedure in women.

**Table 1:** Specificities of imaging modalities and respective advantages/disadvantages

	Specificities in women relevant to the respective imaging modality	Advantages in women	Disadvantages in women
Cardiac CT	- Higher heart rate - Less non-obstructive CAD - Less calcified plaques - Less high-risk plaque features - Smaller diameter of epicardial coronary arteries - Angina for lower degrees of coronary stenosis - FFR-CT higher in women than in men for given stenosis severity	- Calcium scoring: higher sensitivity in women - CCTA: imaging of positive remodeling, a differential diagnosis of non-obstructive CAD - Early detection of plaques and subsequent increase in preventive therapies - Information about plaque composition - Measurement of CT perfusion and FFR-CT - Reduced need for additional testing and costs in women with angina	- Radiation exposure (0.5–7 mSv) - Lower sensitivity and specificity for detection of stable CAD than in men - Lower image quality due to smaller size of epicardial coronary arteries
CMR	- Small left ventricular cavity size in postmenopausal women - T1 and ECV mapping values higher in women than in men - In pregnant women, adapt position to left lateral tilt position	- Devoid of radiation exposure; possible during the 2^nd^ and 3^rd^ trimester of pregnancy - Simultaneous assessment of cardiac volumes, function, and perfusion - Mapping techniques to detect edema and fibrosis - Measurement of GLS to detect CTRCD - Higher sensitivity than SPECT-MPI for stable CAD - Differential diagnosis of MINOCA/INOCA	- Higher rates of side effects of vasodilator agents for stress perfusion CMR - Fetal risk induced by heating effect during 1^st^ trimester of pregnancy - Fetal risk related to gadolinium at any stage of pregnancy - Higher frequency of claustrophobia in women
SPECT	- Small left ventricular cavity size in postmenopausal women - Breast tissue	- High accuracy for detection of myocardial ischemia - Wide availability - If combined SPECT/CT, possible correction of breast attenuation artifacts - If combined SPECT/CT, possible simultaneous quantification of CACS	- Highest radiation exposure of all noninvasive imaging modalities (2–8 mSv) - Higher rates of side effects of vasodilator agents - Small heart artifact - Breast attenuation artifact - No diagnosis of CMVD - Risk of false negatives for small ischemic areas - Underestimation of LVEF value compared to CMR - Excretion of radiotracer in maternal milk: interruption of breastfeeding for > 12 h
PET	- Higher values of MBF at rest - CFR values lower in women than in men	- Reference standard for the quantification of MBF and CFR - High spatial resolution - Correction of breast attenuation artifacts	- Radiation exposure (2–5 mSv) - No routine measurement of cardiac volumes - Excretion of radiotracer in maternal milk: interruption of breastfeeding for > 12 h

Abbreviations. *CACS*: coronary artery calcium score; *CAD*: coronary artery disease; *CCTA*: coronary computed tomography angiography; *CMR*: cardiac magnetic resonance; *CMVD*: coronary microvascular dysfunction; *CFR*: coronary flow reserve; *CT*: computed tomography; *CTRCD*: cancer treatment-related cardiac dysfunction; *ECV*: extracellular volume; *FFR*: fractional flow reserve; *GLS*: global longitudinal strain; *INOCA*: ischemia with no obstructive coronary artery disease; *LVEF*: left ventricular ejection fraction; *MBF*: myocardial blood flow; *mSv*: milliSievert; *MINOCA*: myocardial infarction with no obstructive coronary artery disease; *MPI*: myocardial perfusion imaging; *PET*: positron emission tomography; *SPECT*: single-photon emission computed tomography

In this review, we summarize the main female characteristics in pathophysiology and clinical presentation of the most frequent cardiovascular conditions and discuss the contemporary limitations of cardiac imaging in women. We further present four clinical scenarios, including seven case examples, where cardiac imaging proved useful in women with suspected or manifest CVD.

## Pathophysiological features of cardiovascular diseases in women

The most obvious pathophysiological differences between women and men in relation to CVD are linked to sex hormones. Relatively protected against CVD before menopause, women’s risk exceeds men’s risk after menopause, highlighting the cardioprotective influence of sex hormones, particularly estrogens [2]. Conversely, female-specific diseases associated with dysregulation of sex hormones, such as polycystic ovary syndrome and premature menopause, increase cardiovascular risk [3].

Mutiple pathophysiological mechanisms are shared between both sexes but display a sexual dimorphism resulting in different phenotypes of CVD. Coronary microvascular dysfunction (CMVD) [4] is a condition of microvessel impairment leading to myocardial ischemia even in the absence of epicardial coronary artery stenosis [5]. Several sex-specific biological, hormonal, and neurological pathways promote CMVD, acting in isolation or synergistically [6]. Indeed, CMVD is favored by low-grade systemic inflammation and increased sympathetic activity, which are more pronounced in women compared to men, as well as by the decrease of estrogens in postmenopausal women [7–9]. Importantly, CMVD is thought to be the *common soil* of various CVDs affecting most frequently postmenopausal women, such as ischemia with no obstructive coronary artery disease (INOCA), heart failure (HF) with preserved ejection fraction (HFpEF), Takotsubo cardiomyopathy (TTC, also termed stress-induced cardiomyopathy, apical ballooning syndrome or broken-heart-syndrome), peripartum cardiomyopathy (PPCM), and cardiomyopathy related to antineoplastic treatments [10–12], all of which will be discussed in this review.

Negative emotions can also trigger CVD via the so-called brain–heart axis [13, 14]. An elevated amygdalar metabolic activity, a brain region involved in the processing of emotions, is associated with an increased risk of future major adverse cardiovascular events (MACE) [15]. In women, but not in men, an association between the presence of myocardial ischemia and an increased amygdalar metabolic activity has recently been shown [16] and is consistent with a high prevalence of mental stress in women with CVD [13]. Similarly, women are at a higher risk of mental stress-induced myocardial ischemia than men [17], which might be associated with the increased baseline sympathetic activity in older women [18]. Sympathetic hypertonia also plays a detrimental role in HF [19] and TTC [20] and may account, at least in part, for the gender bias and sex-specific phenotypes seen in these conditions.

## Sex differences in cardiovascular diseases and their impact on cardiac imaging

### *Coronaryartery disease in women *

Coronary artery disease (CAD) differs between women and men in terms of risk factors—with a higher impact of traditional cardiovascular risk factors (CVRFs) in women, despite a lower overall risk burden [21], clinical presentation—more often atypical in women [3], mechanisms—with lower atherosclerotic plaque burden in women [22], and outcomes—worse prognosis in women, despite lower CAD burden [23]. In addition, women more frequently report non-traditional CVRFs, such as mental stress and depression [13]. Mechanistically, plaque composition differs between sexes with women presenting more often with plaque erosion during an acute coronary syndrome (ACS) (as compared to plaque rupture in men), less necrotic core, and less plaque calcification [24]. These sex differences in plaque composition could account for the higher prevalence of ischemia with non-obstructive CAD in women [24], a central feature in the female population of both acute and chronic coronary syndromes (CCS). Consequently, the ongoing paradigm that CAD imaging consists of detecting epicardial coronary stenosis must be reconsidered in women [24].

**Fig. 1:**
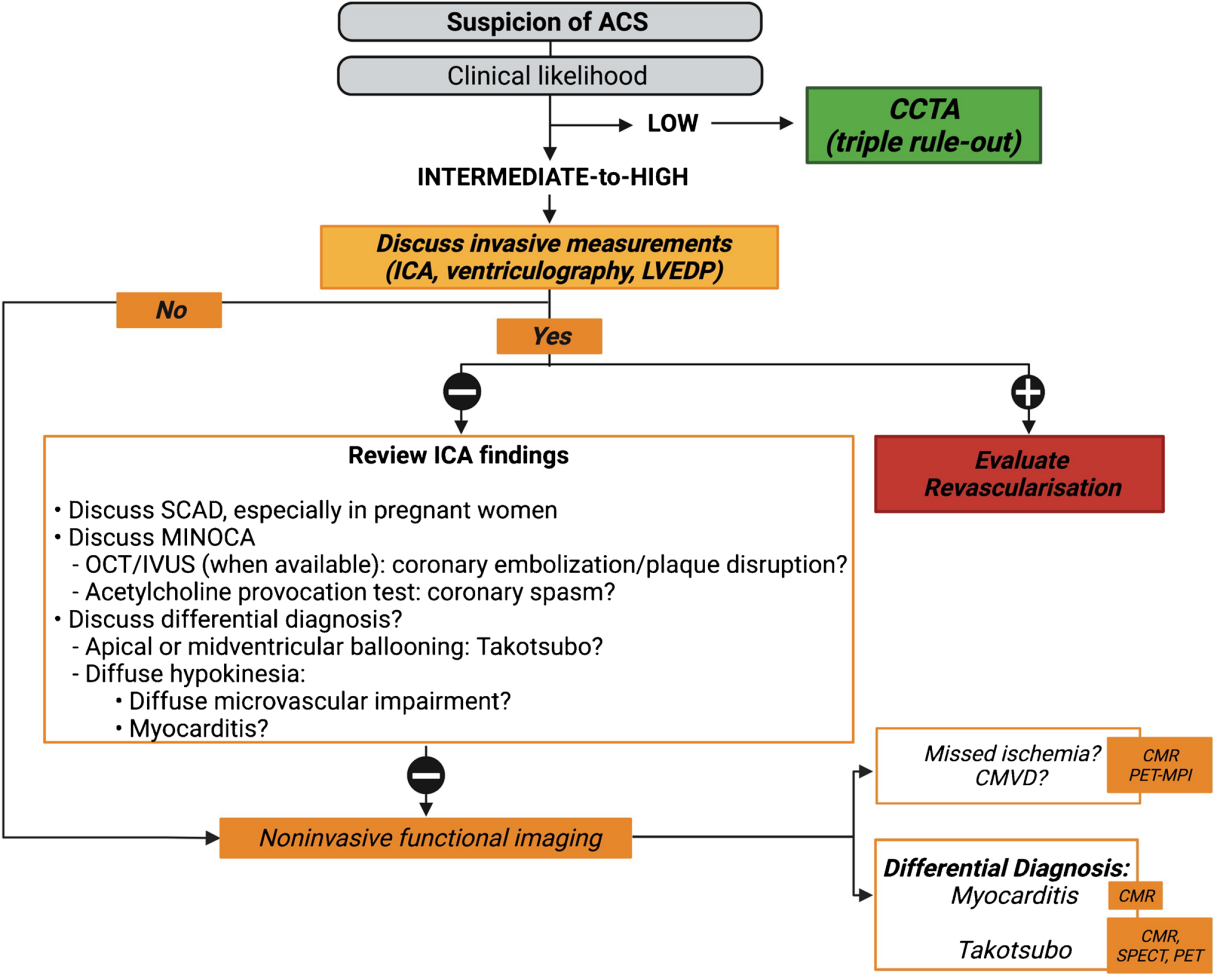
Proposed diagnostic algorithm for acute coronary syndrome. In case a STEMI is suspected, urgent ICA is recommended [235]. In case a NSTEMI is suspected, the diagnostic approach depends on the clinical likelihood of CAD, ECG findings, and troponin measurement [25]. If the clinical likelihood is low, ECG-triggered contrast-enhanced CT can rule out simultaneously coronary stenosis, aortic dissection, and pulmonary embolism (triple rule-out) [25]. If the clinical likelihood is intermediate-to-high, ICA must be discussed, urgently or semi-urgently. If no coronary stenosis is found on ICA, MINOCA should be suspected. A first step consists of thoroughly reviewing the ICA to search for subtle signs of SCAD, coronary embolization, or plaque disruption, using IVUS or OCT, when available [32, 236]. After symptom resolution and exclusion of other causes, invasive provocative testing using acetylcholine, ergonovine, or methylergonovine can help to establish a definitive diagnosis. Nevertheless, it should be used with caution and only by experienced operators, and in all cases never in the acute setting of the episode [29]. LV angiography can also provide important information such as segmental hypokinesia suggesting epicardial abnormality, apical or midventricular ballooning being in favor of TTC cardiomyopathy, and a more diffuse hypokinesia sometimes suggesting a microvascular impairment [237]. If a diagnosis cannot be established, advanced noninvasive imaging is required. CMR can show patterns of ischemia/infarct, evidence MINOCA causes [82, 83], and rule out differential diagnosis such as TTC and myocarditis [26, 30, 83]. MPI can also be used in the acute/subacute setting, after symptom resolution and normalization of ECG and troponin [238]. **Abbreviations**: *ACS*: acute coronary syndrome;* CAD*: coronary artery diseases; *CMR*: cardiac magnetic resonance; *CCTA*: coronary computed tomography angiography; *CT*: computed tomography; *ECG*: electrocardiogram; *ICA*: invasive coronary angiography; *IVUS*: intravascular ultrasound ; *LV*: left ventricle; *LVEDP*: left ventricular end-diastolic pressure; *MI*: myocardial infarction; *MINOCA*: myocardial infarction with no obstructive coronary artery disease; *MPI*: myocardial perfusion imaging; *N*: normal; *NSTEMI*: non ST-elevation myocardial infarction; *OCT*: optical coherence tomography; *PET*: positron emission tomography; *SCAD*: spontaneous coronary artery dissection; *SPECT*: single-photon emission computed tomography; *STEMI*: ST-elevation myocardial infarction; *TTC*: Takotsubo cardiomyopathy

In ACS, the majority of cases occur due to a plaque rupture which leads to a coronary occlusion, and is more frequent in men [25]. However, a subgroup of individuals displays myocardial infarction (MI) with no obstructive coronary arteries (MINOCA), of which the majority are women [26, 27]. MINOCA is defined as (i) an acute MI (as per the 4th universal definition) [28], (ii) with no obstructive coronary arteries on invasive coronary angiography (ICA), (iii) and no specific differential diagnosis, which requires excluding myocarditis and TTC [29, 30]. While MINOCA remains of unknown origin in 8–25% of cases [30], it can also be induced by specific conditions with high female prevalence, including coronary spasm (Case 1) and spontaneous coronary artery dissection (SCAD, see the “Specific cardiac diseases in pregnancy” section) [31]. Spontaneously resolving coronary plaque erosion can also cause MINOCA [32]. Given the specific etiologies of ACS in women, a new classification has been proposed in this population. Indeed, using the universal definition of MI, 1 out of 8 young women (< 55 years) with ACS remains unclassified [33]. The VIRGO (Variation in Recovery: Role of Gender on Outcomes of Young AMI Patients) classification, which groups patients according to their clinical features, reduces the rate of unclassified cases thereby helping to tailor management strategies [34] (Fig. 1).

**Fig. 2:**
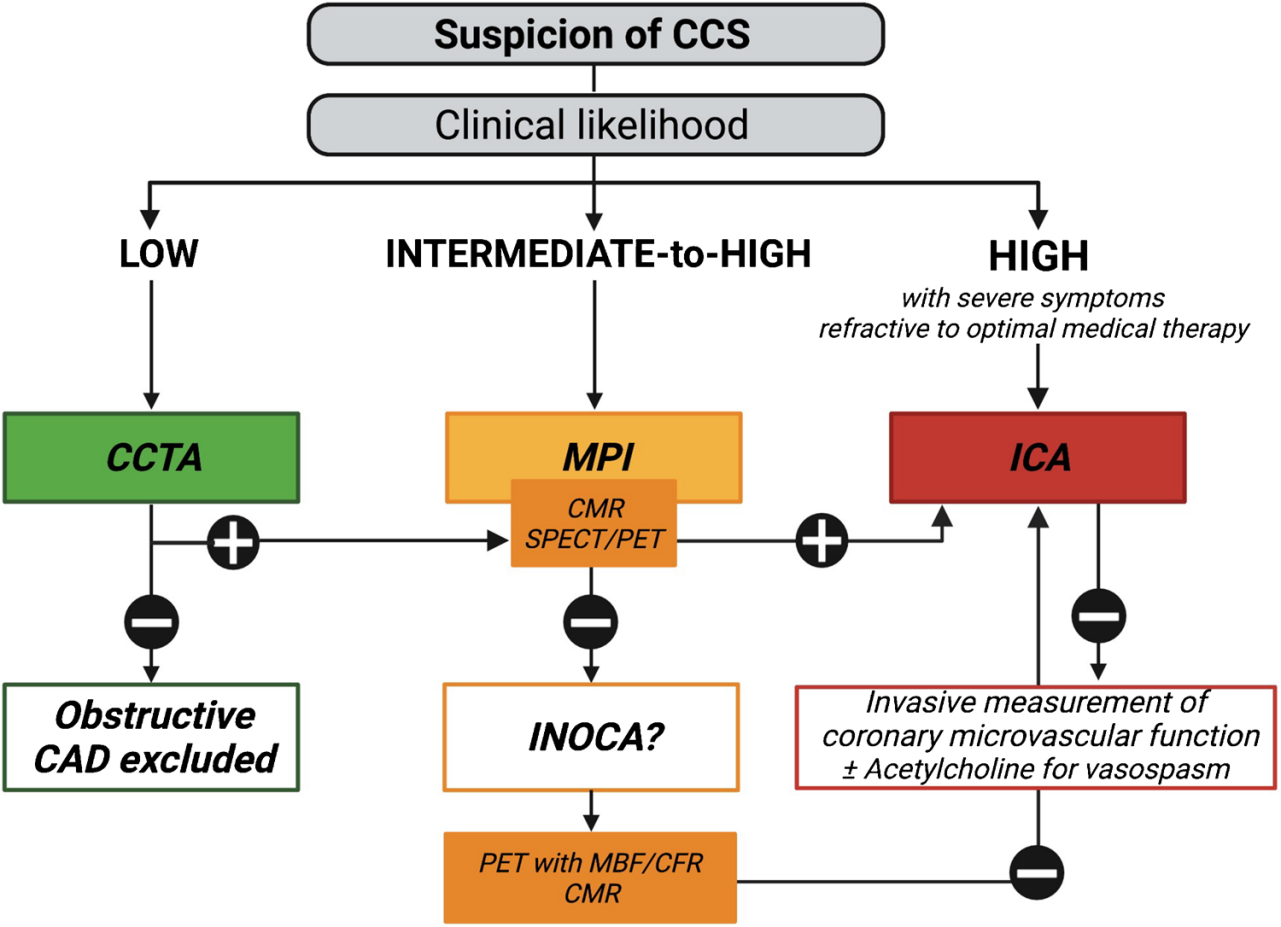
Proposed diagnostic algorithm for chronic coronary syndrome. In case of suspected CCS, the choice of the imaging modality also depends on the clinical likelihood of CAD, which varies according to sex. Indeed, for a similar clinical presentation and age, the clinical likelihood is usually lower in women than in men [239]. If the clinical likelihood is low (< 15%), CCTA safely rules out obstructive CAD [240]. If it is intermediate-to-high (15–85%) or in case of low likelihood, but persistent symptoms, exercise ECG or stress echo are recommended as an initial test [241]. However, pretest risk assessment in women is limited because of frequently atypical symptoms [242] and lower performances of ECG stress tests [243], related to a high rate of false positives (due to the higher prevalence of non-obstructive CAD in women) and to lower maximal exercise capacities than men [244]. Women also present a higher prevalence of concave-shaped chest wall than men, which is associated with increased false positive stress echocardiography findings [128]. Consequently, noninvasive MPI (SPECT, CMR) is preferred. Based on the findings of the latter, ICA can be discussed. If ischemia or coronary stenosis cannot be detected but symptoms persist and the suspicion of CAD remains high, INOCA must be considered in women. PET-MPI with calculation of MBF and CFR is the preferred noninvasive modality. Alternatively, CMR can be used. If noninvasive MPI does not allow ruling out INOCA, or if ICA is negative, invasive measurement of coronary microvascular function (IMR, CFR, and/or FFR) can be discussed, as well as vasoreactivity testing with acetylcholine [245]. **Abbreviations**: *CAD*: coronary artery disease; *CCS*: chronic coronary syndrome; *CCTA*: coronary computed tomography angiography; *CFR:* coronary flow reserve; *CMR*: cardiac magnetic resonance; *ECG*: electrocardiogram; *FFR*: fractional flow reserve; *ICA*: invasive coronary angiography; *IMR*: index of microvasculature resistance; *INOCA*: ischemia with no obstructive coronary artery disease; *MBF*: myocardial blood flow; *MPI*: myocardial perfusion imaging; *PET*: positron emission tomography; *SPECT*: single-photon emission computed tomography

Similarly, in CCS, more women than men present with symptoms of myocardial ischemia but no obstructive coronary arteries (INOCA) [35]. INOCA is the clinical manifestation of two different mechanisms that can overlap, i.e., CMVD (Case 2) and vasospastic angina [35]. One particular form of INOCA with a female overrepresentation is mental stress-induced myocardial ischemia [17], which consists of a left ventricular ejection fraction (LVEF) decline or a new regional wall motion abnormality or a perfusion decrease following mental stress [36]. As INOCA is associated with an increased risk of MACE [37], which is usually not captured by traditional risk scores [38], the diagnostic strategy must be adapted in the female population (Fig. 2).

### Specific considerations of imaging modalities for coronary syndromes in women

Advanced noninvasive cardiac imaging plays a critical role in women with suspected CAD [39] (Table 2). The most commonly used techniques are coronary computed tomography angiography (CCTA), cardiac magnetic resonance (CMR) imaging and myocardial perfusion imaging (MPI), i.e., ^99m^Technetium (^99m^Tc) and ^201^Thallium (^201^Tl) single-photon emission computed tomography (SPECT), and positron emission tomography (PET) using ^82^Rubidium (^82^Rb), ^13^ N-ammonia (^13^N-NH_3_), or ^15^O-water (^15^O) [40].

**Table 2:** Imaging findings of diseases of specific interest in women

	Specificities in women relevant to the respective imaging modality	Noninvasive imaging tools	Imaging findings
Ischemic heart disease	MINOCA	- SPECT (^201^Tl, ^99m^Tc) and PET (^82^Rb, ^13^N-NH_3_, ^15^O-H_2_O) perfusion tracers	- Myocardial fixed perfusion defect in case of myocardial necrosis
		- CMR	- Myocardial fixed perfusion defect in case of myocardial necrosis - Subendocardial edema and LGE in case of ischemia
		- CCTA	- Non-obstructive CAD, positive remodeling
	Microvessel disease	- PET perfusion tracers (^82^Rb, ^13^N-NH_3_, ^15^O-H_2_O)	- Absence of segmental perfusion defect with a distribution suggestive of epicardial origin - MBF < 1.8 mL/g/min for ^13^N-NH_3_, < 2.3 mL/g/min for ^15^O-H_2_O, no standard threshold for ^82^Rb - CFR < 2.0 for ^82^Rb and for ^13^N-NH_3_, < 2.5 for ^15^O-H_2_O
		- CMR (not used in clinical routine)	- Reduced MPR < 2.2 - Absence of segmental perfusion defect with vascular distribution
		- CCTA	- Absence of obstructive CAD
Heart failure	HFpEF	- CMR	- LVEF ≥ 50%, non-dilated LV, concentric hypertrophy, and left atrial enlargement
		- SPECT and PET radiotracers	- No specific finding
		- CCTA	- No specific finding
	TTC	- SPECT (^99m^Tc, ^201^Tl) and PET (^82^Rb, ^13^N-NH_3_, ^15^O-H_2_O) perfusion radiotracers - Glucose metabolism (^18^F-FDG) - Myocardial sympathetic innervation (^123^I-MIBG)	- Reduced perfusion in the acute phase, normalized perfusion in the subacute phase - Reduced in the acute and subacute phase - Reduced in the acute and subacute phase
		- CMR	- Kinetic abnormalities (apical, basal, or midventricular), myocardial edema, LV thrombi in case of complication - No sign of necrosis
		- CCTA	- Absence of obstructive CAD

Abbreviations. ^*13*^*N-NH*_*3*_: nitrogen-13 radiolabelled ammonia; ^*15*^*O-H*_*2*_O: oxygen-15 radiolabeled water; ^*18*^*F-FDG*: fluor-18 radiolabeled fluorodeoxyglucose; ^*82*^*Rb*: Rubidium-82; ^*123*^*I-MIBG*: iodine-123-meta-iodobenzylguanidine; ^*99m*^*Tc*: technetium-99 m; ^*201*^*Tl*: Thallium-201; *CAD*: coronary artery disease; *CCTA*: coronary computed tomography angiography; *CFR*: coronary flow reserve; *CMR*: cardiac magnetic resonance imaging; *HFpEF*: heart failure with preserved ejection fraction; *LGE*: late gadolinium enhancement; *LV*: left ventricle; *LVEF*: left ventricle ejection fraction; *MBF*: myocardial blood flow; *MINOCA*: myocardial infarction and no obstructive coronary artery disease; *MPR*: myocardial perfusion reserve; *PET*: positron emission tomography; *SPECT*: single-photon emission computed tomography; *TTC*: Takotsubo cardiomyopathy

#### Coronary computed tomography angiography

CCTA allows the detection and assessment of coronary stenosis severity, with obstructive CAD being defined as a stenosis ≥ 70%, or ≥ 50% if ischemia is documented [41]. Despite an overall high diagnostic accuracy for the detection of stable CAD [40, 42], the sensitivity and specificity of CCTA is slightly lower in women than in men [43, 44], owing to the smaller diameter of coronary vessels in women as well as to their frequently higher heart rates resulting in motion artifacts and lower image quality [45]. Women with moderate or severe ischemia are more likely to have non-obstructive CAD (i.e., < 50% stenosis in all vessels) on CCTA than men [46], and they present angina symptoms for lower degrees of stenosis than men [47]. This could again be explained by the fact that women have smaller epicardial coronary arteries [48] and more often positive remodeling, which might become symptomatic at lower degrees of stenosis than in men [47], particularly in distal segments and side branches [44]. Additionally, the coronary diameter is a key parameter predicting image quality of CCTA examination, which is therefore more frequently lower in women than in men [49]. Nevertheless, CCTA results in lower use of additional testing and costs in women than in men, although at the expense of higher radiation exposure compared to functional testing [50].

Beyond detection of coronary stenosis, CCTA informs about plaque composition, which is of prognostic value regardless of the degree of stenosis. Indeed, evaluation of low-attenuation plaque volume (< 30 Hounsfield units) is a better predictor of future ACS than plaque calcification and stenosis severity [51]. This could be particularly interesting in women who usually exhibit lower plaque burden and calcifications than men [52], notwithstanding worse long-term outcomes [23]. Although women display less high-risk plaque features than men (low-attenuation plaques, positive remodeling, spotty calcifications, and napkin ring sign) [53], a study in 4415 patients with stable chest pain without known CAD showed that high-risk plaques found by CCTA were a stronger predictor of MACE in women than in men [54]. While calcified plaques are usually non-modifiable, the progression of non-calcified plaques can potentially be modified by preventive treatment such as statins [55]. Accordingly, the early detection of plaque progression with CCTA could result in an early initiation of treatment, which may improve patients’ outcomes [56]. Moreover, plaque regression under preventive treatment, as assessed by CCTA, is a promising imaging biomarker of treatment efficacy that may help tailor the therapeutic strategy [57].

Non-contrast cardiac computed tomography (CT), whose effective radiation dose amounts to < 1 mSv using contemporary scanners [58], can also provide valuable information about the calcified plaque burden in coronary arteries (Agatston coronary artery calcium score, CACS), a powerful predictor of subsequent MACE [59]. Although overall coronary calcium burden is lower in women than in men, CACS is a better predictor of MACE in the female population than in men, with a similar burden of calcified plaques being associated with a worse prognosis in women [60]. However, CACS does not rule out non-calcified plaques, stressing the importance of plaque composition analysis in women, in whom calcifications appear nearly 10 years later than in men [47]. Nevertheless, when matching the baseline plaque volume, the progression rate of plaque burden appears mediated mainly by calcified plaques in women and by non-calcified plaques in men [61]. Therefore, a careful evaluation of changes in plaque composition could help fine-tune the coronary risk stratification, especially in the female population [57]. Noteworthy, breast arterial calcifications are associated with subclinical CAD and their detection might improve risk stratification in asymptomatic women [62].

Additional novel CCTA tools have emerged lately, which could improve the detection of ischemia in women [63]. Indeed, it has been shown that adding CT perfusion to CCTA improves the specificity of ischemia detection compared to CCTA alone in women, but not in men [64]. CT-derived fractional flow reserve (FFR-CT) was shown to be higher in women than men regardless of stenosis degree, and women tend to have a lower likelihood of positive FFR-CT than men for a similar degree of coronary stenosis [65]. This highlights the need for sex-based thresholds and further studies exploring the incremental prognostic value of FFR-CT in women.

The use of CCTA remains limited in young women due to breast radiation exposure and in pregnant women, with estimated radiation of 0.5–7 mSv [66]. For a similar exposure from cardiac imaging, the risk of developing cancer is higher in women than in men, which could be related to relatively smaller body sizes in women [67]. Nevertheless, technological advances and refinements of the scanning protocol may allow a significant reduction of the effective breast tissue dose [68].

#### Cardiac magnetic resonance

CMR is particularly interesting in women with suspected CAD because it is devoid of ionizing radiation exposure [69]. In women with stable CAD, CMR has a higher sensitivity than SPECT as demonstrated by the CE-MARC [70] and MR-IMPACT II studies [71]. Two CMR techniques are available to detect myocardial ischemia [72]: perfusion CMR using a vasodilator agent and requiring a gadolinium-based contrast agent to assess perfusion abnormalities, and dobutamine-stress CMR, which focuses on wall motion abnormalities and therefore does not require contrast agents. Noteworthy, the higher rate of side effects induced by vasodilator agents in women (such as headache, flushes, dizziness, chest pain, and nausea), in particular with adenosine [73], represents a limitation to their use. Interestingly, first-pass myocardial perfusion on vasodilator stress CMR can detect subendocardial ischemia even in the absence of significant coronary stenosis, a frequent presentation in women [30]. Although not yet implemented in clinical routine, quantification of myocardial blood flow (MBF) by rest/stress high-resolution perfusion CMR displays good accuracy to identify CMVD [74]. CMR’s high spatial resolution can also be useful in the follow-up of women with ACS undergoing revascularization, who present with smaller post-revascularization infarct size and myocardial salvage than men [75]. T1 and T2 mapping techniques and CMR-based measurement of extracellular volume (ECV) help to accurately assess the acute infarct size [76]. T1 mapping is highly sensitive to edema (such as in the acute phase of MI [77]), and fibrosis (such as in scarred myocardium following MI [78]). ECV measurement using mapping techniques also detects myocardial fibrosis with high sensitivity, complementing the data obtained from T1 mapping [79]. While late gadolinium enhancement (LGE) only highlights focal fibrosis [80], T1 and ECV mapping allow the detection of diffuse interstitial fibrosis, even in the early phases of the disease [81]. Similarly, T2 myocardial maps can be generated, which, if increased, indicate myocardial edema [82]. CMR mapping techniques simultaneously assess differential diagnoses of CAD in women such as myocarditis and TTC [83]. Indeed, early CMR imaging with T1 and ECV mapping techniques significantly improves the detection of acute phases of TTC compared to CMR without mapping, by evidencing myocardial edema in the affected area [83, 84].

Noteworthy, T1 and ECV values are higher in women than in men [85]. Additionally, T1 and ECV increase with age in males, but not in females [79], which could reflect the age-dependent increase of interstitial myocardial fibrosis in males observed in histopathological studies [86]. While these differences might be irrelevant in diseases inducing high levels of myocardial edema and/or fibrosis, they could become significant in diseases inducing mild changes, suggesting the need for sex-specific reference values for mapping parameters [79]. Another advantage of CMR in women as compared to SPECT is that CMR is devoid of breast attenuation artifacts [87]. Moreover, CMR is not associated with radiation exposure and is therefore encouraged over ionizing techniques (SPECT, PET) in premenopausal women with intermediate-to-high risk of CAD [88]. Nonetheless, a limitation of CMR in women is their higher rate of claustrophobia [89].

#### Nuclear imaging modalities

SPECT is the most common functional imaging technique for ischemia detection and risk stratification of women with stable CAD [90]. Despite overall high diagnostic accuracy in women [91], the detection of small areas of ischemia by SPECT-MPI can be limited in postmenopausal women who present with smaller left ventricular (LV) volumes, which in conjunction with the limited spatial resolution of SPECT induce image blurring [90]. In addition, SPECT-MPI cannot rule out CMVD [92] as it does not enable absolute quantification of MBF due to its low resolution. Another limitation of SPECT in women is breast tissue attenuation which can mimic perfusion defects, classically in the anterior wall [93]. Several techniques help to reduce attenuation artifacts including image acquisition in prone position, breast bandage, and CT-based attenuation correction [94]. Combining CT with SPECT also allows quantifying intrathoracic fat and CACS, which provide incremental prognostic value for MACE in women [95].

Radiation exposure represents a major drawback of SPECT-MPI in young women [96], with an estimated exposure of 2–8 mSv [66]. ^99m^Tc should therefore be favored over ^201^Tl because of its lower radiation exposure [88]. Additionally, if stress imaging is normal, skipping rest acquisitions can decrease total radiation dose [97]. Highly sensitive cadmium-zinc-telluride-based detectors also allow the reduction of the amount of injected radiotracer and the associated radiation burden [98] while improving diagnostic accuracy in women with low-to-intermediate likelihood of CAD. Indeed, cadmium-zinc-telluride-based detectors reduce the percentage of artefactual perfusion defects, in particular those induced in the anterior wall by breast attenuation [99]. Similarly, PET-MPI is associated with a lower radiation exposure than SPECT [100]. Additionally, PET’s higher spatial resolution compared to SPECT reduces the rate of false negatives in women that are related to smaller LV size [90]. Moreover, PET-MPI (using either ^13^N-NH_3_, ^82^Rb, or ^15^O-H_2_O) enables absolute quantification of myocardial perfusion, i.e., MBF and coronary flow reserve (CFR), which are key elements of CMVD [101]. Of note, MBF values at rest are higher and CFR values are lower in women than in men [102], and the predictive value of PET-derived MBF and CFR for MACE is lower in women than in men [103, 104]. On the other hand, a blunted heart rate response after pharmacological stress for ^13^N-NH_3_-PET-MPI is a predictor of reduced CFR in women with suspected myocardial ischemia, but not in men [105]. More recently, ^18^F-flurpiridaz has emerged as a promising tracer for PET-MPI evaluation of MBF [106]. ^18^F-flurpiridaz-PET-MPI presents higher specificity than SPECT-MPI for the detection of CAD in women [107], which could be related to the preserved diagnostic performances of ^18^F-flurpiridaz-PET-MPI in patients with small ventricles [108]. Similar to SPECT-MPI, CT performed in combination with PET-MPI also helps correct breast attenuation artifacts [109].

With regard to plaque imaging, several studies have established the ability of vascular ^18^F-sodium fluoride PET (^18^F-NaF PET) to document the early stages of plaque microcalcification [110] and to predict the progression of coronary plaques [111]. Interestingly, the intensity of ^18^F-NaF uptake in atherosclerotic plaques of CVD-free patients is lower in women than in men [112], which seems consistent with the lower burden of calcified plaque in this population. Consequently, studies are needed to identify specific features of plaque progression other than calcification in the female population.

### Heart failure in women

The presentation of HF can be acute or chronic. Three types of chronic HF are distinguished based on LV ejection fraction (LVEF): HF with reduced LVEF (≤ 40%, HFrEF), HF with mildly reduced LVEF (41–49%, HFmrEF, previously HF with midrange reduced EF), and HFpEF (≥ 50%) [113, 114]. Women in general display a higher LVEF than men, this difference becoming more pronounced with age. Indeed, a substantial decrease in LV end-systolic volume in aging women accounts for a concomitant increase in LVEF [113, 115]. It is therefore conceivable that women with 51% ≤ LVEF ≤ 59% in fact present early stages of HFrEF [116] and are wrongly categorized as HFpEF [113]. Interestingly, supra-normal LVEF (≥ 65%) is associated with an increased risk of death in women, but not in men [117, 118].

In Western countries, HFpEF affects 5% of the population aged ≥ 60 years and represents more than half of all HF-related hospitalizations [119]. HFpEF displays a clear female overrepresentation with a ~ 2:1 ratio [12]. Besides female sex, risk factors for HFpEF include advanced age > 70 years, metabolic syndrome, and atrial fibrillation [120]. All these factors induce a systemic proinflammatory state, which favors CMVD. The latter is accompanied by a reduction of nitric oxide and G protein activities, which triggers myocardial hypertrophy and fibrosis, and in turn LV diastolic dysfunction [121]. The central role of CMVD in the pathophysiology of HFpEF could explain the female overrepresentation in this entity [6]. HFpEF can also be the manifestation of specific cardiomyopathies, such as inherited or acquired infiltrative cardiomyopathies (including cardiac amyloidosis), restrictive cardiomyopathies, myocarditis, or genetic cardiomyopathies [120]. Notably, women with HFpEF have a better prognosis than men with HFpEF, with less mortality despite a higher re-hospitalization rate, which could possibly reflect a sex difference in spironolactone treatment impact on all-cause mortality [122].

HFrEF in women is less frequently of ischemic origin than in men; and if so, it is more often related to CMVD than to epicardial stenosis [12]. A specific cause of acute HFrEF with female overrepresentation includes TTC-related acute HF [123]. TTC consists of acute and transient systolic LV dysfunction developing in the direct aftermath of emotional or physical stress, though sometimes no clear trigger can be identified [123]. Given its association with emotional stress, neurogenic myocardial stunning mediated by stress-induced catecholamine release has been suggested to be the most likely causative mechanism of TTC. Nevertheless, multivessel coronary spasms, impaired coronary microcirculation, or inflammatory processes have been proposed as alternative mechanisms [124]. TTC is predominantly a female postmenopausal disease with women representing 90% of cases of which 80% are diagnosed after the age of 50 [123]. TTC classically mimics ACS with ST-elevation and increased troponin; hence, TTC is a differential diagnosis of MINOCA [28]. TTC can also present as acute HF or less frequently be asymptomatic [123]. The hallmark feature of TTC is a reversible LV apical ballooning, although inverted midventricular, basal, and focal forms have also been described [123].

Other female-associated causes of acute HF are breast cancer treatment-related cardiomyopathy, and female-specific cardiomyopathies such as PPCM [12], which will be discussed in the respective sections of this review.

**Fig. 3:**
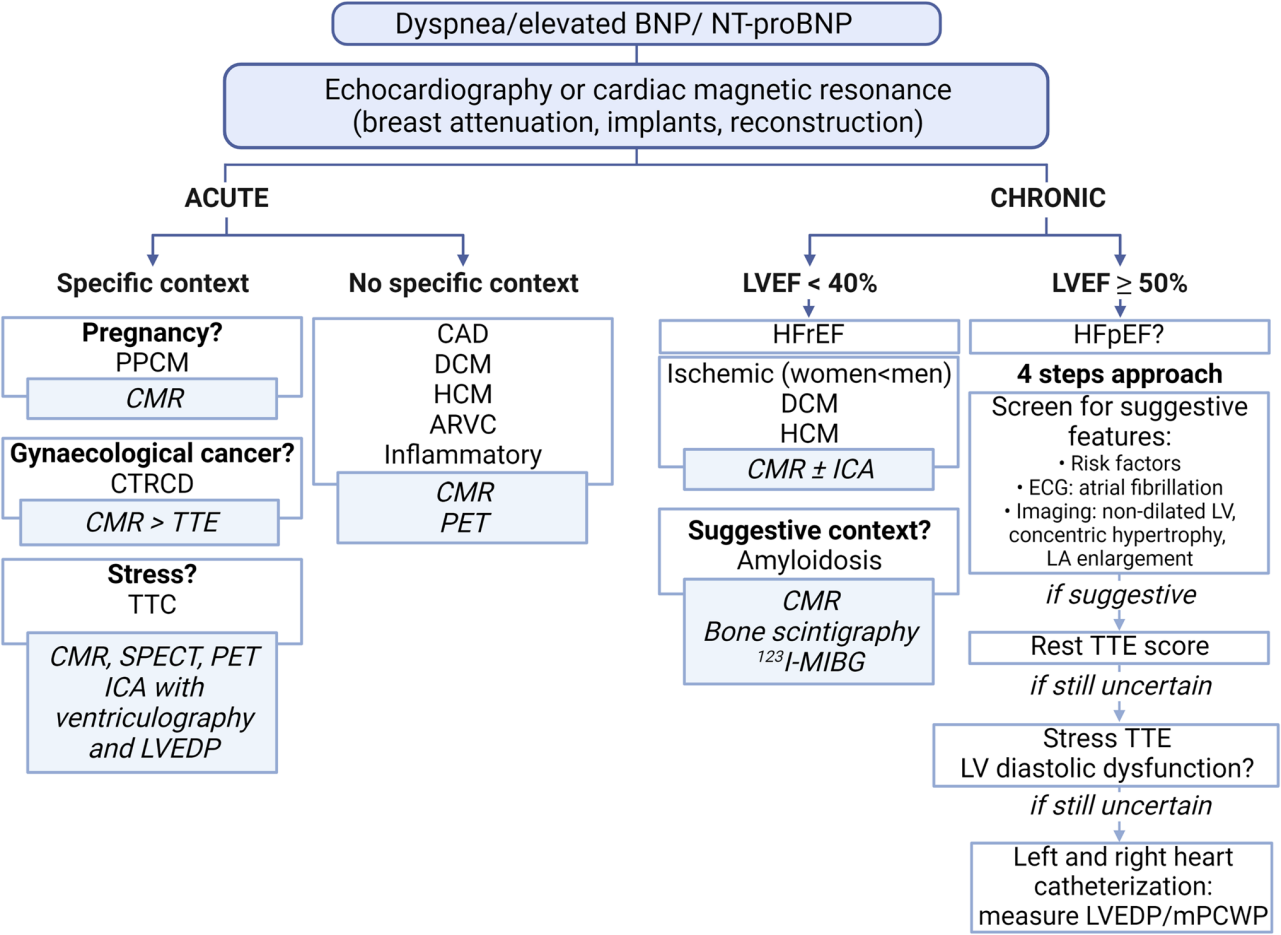
Proposed diagnostic algorithm for women with suspected heart failure. In both acute and chronic HF, TTE is the frontline test for the evaluation of cardiac contractility and chamber volumes, in addition to cardiac biomarkers (troponin and brain natriuretic peptide) [114]. Because of TTE’s potential limitations in women, CMR is a useful alternative [127] to assess systolic and diastolic function and determine the type of HF. In chronic HF, ERNA is another option [132, 136], although no longer mentioned in the latest European Society of Cardiology guidelines [114]. In case of HFrEF, the lower rate of ischemic origin in women stresses the importance of CMR with LGE to evaluate alternative etiologies, such as DCM, HCM, VHD [142], ARVC [144], myocarditis, sarcoidosis, and infiltrative diseases. CCTA is also well suited to rule out CAD in women with HFrEF given its high specificity [47]. In selected cardiomyopathies, nuclear imaging is useful for the etiological workup, such as bone scintigraphy and ^123^I-MIBG SPECT for cardiac amyloidosis [140], ^18^F-FDG PET in inflammatory diseases, and nuclear MPI, ^123^I-MIBG SPECT, and ^18^F-FDG PET in TTC [147]. In women with dyspnea and preserved systolic LVEF, an in-depth screening for HFpEF must be considered, based on the four-step algorithm previously mentioned and established by the ESC [120, 130]. **Abbreviations**: ^18^*F-FDG*: Fluor-18-fluorodeoxyglucose; ^123^*I-MIBG*: iodine-123-meta-iodobenzylguanidine; *ARVC*: arrhythmogenic right ventricular cardiomyopathy; *CAD*: coronary artery disease; *CCTA*: coronary computed tomography angiography; *CAD* *CMR*: cardiac magnetic resonance; *CTRCD*: cancer therapy-related cardiac dysfunction; *DCM*: dilated cardiomyopathy; *ERNA*: equilibrium radionuclide angiocardiography; *HCM*: hypertrophic cardiomyopathy; *HF*: heart failure; *HFpEF*: heart failure with preserved ejection fraction; *HFrEF*: heart failure with reduced ejection fraction; *ICA*: invasive coronary angiography;* LA*: left atrium; *LGE*: late gadolinium enhancement; *LV*: left ventricular; *LVEDP*: left ventricular end-diastolic pressure; *LVEF*: left ventricular ejection fraction; *mPCWP*: mean pulmonary capillary wedge pressure; *MPI*: myocardial perfusion imaging; *PET*: positron emission tomography; *PPCM*: peripartum cardiomyopathy; *SPECT*: single-photon emission computed tomography; *TTC*: Takotsubo cardiomyopathy; *TTE*: transthoracic echocardiography; *VHD*: valvular heart disease

Noninvasive imaging plays a key role in the management of both acute and chronic HF [125, 126] (Table 2). Although the general diagnostic strategy is similar between both sexes, certain aspects need to be considered in women, related either to the choice of imaging modality to assess cardiac function, or to the etiologies of HF (Fig. 3).

### Imaging-related specificities in heart failure

#### Assessment of cardiac function and volumes

While echocardiography is the first-line exam to assess cardiac function [127], it may be technically limited in women because of a smaller acoustic window related to breast interference and higher prevalence of a concave-shaped chest wall in women [128]. CMR is therefore a valid alternative to echocardiography for imaging of cardiac volumes [125]. CMR is considered the reference standard to measure right and left chamber volumes and mass [125], to assess both systolic and diastolic functions, determine HF etiology, and look for complications in HFrEF [129] as well as in HFpEF [130]. Noteworthy, sex differences exist in the normal values of cardiac chambers, with lower left and right ventricular and atrial volumes as well as LV mass in women as compared to men, even after correcting for body surface area [131].

Based on local practices and availability of technologies, an alternative method is 2D-gated equilibrium radionuclide angiocardiography (ERNA), which robustly determines LVEF [132]. However, scintigraphy tends to underestimate the value of LVEF compared to CMR in both sexes [133].

CCTA also accurately assesses LVEF [134]. Despite this, concerns related to radiation exposure in women limit the use of CCTA and ERNA to patients with low echogenicity and contraindications to CMR [135, 136]. Also, due to the increasing use of prospective electrocardiogram (ECG) gating, which has resulted in lower radiation dose and better image quality [137], assessment of LV volumes by CCTA is no longer ubiquitously available.

**Case 1:**
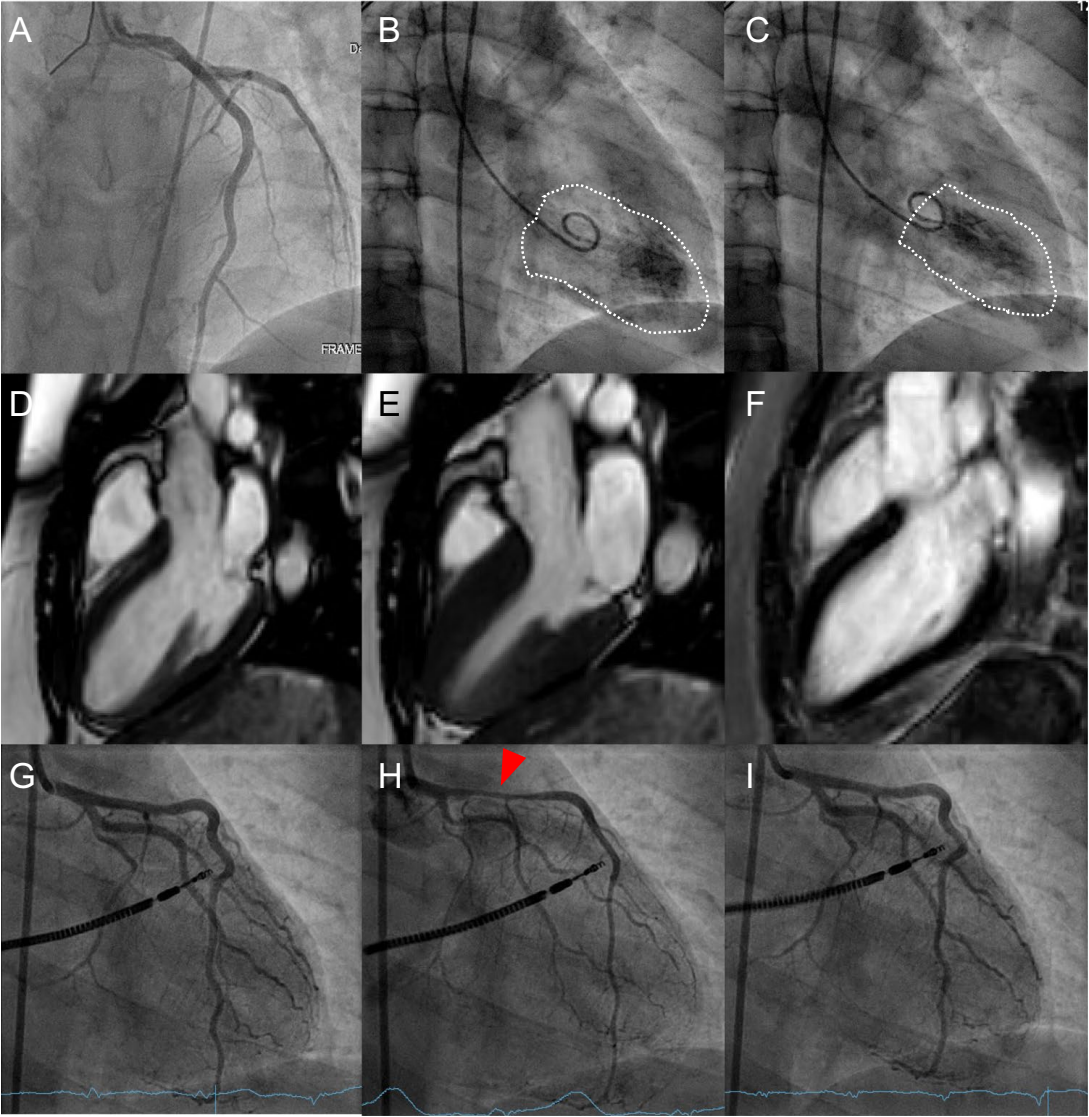
Cardiac arrest in a 28-year-old woman. A 28-year-old woman with no medical history other than smoking was admitted to the intensive care unit after an out-of-hospital cardiac arrest with ventricular fibrillation on ECG, return of spontaneous circulation after 8 min of cardiopulmonary resuscitation and external defibrillation. At admission, ECG revealed ST-elevation in the anterior leads. Echocardiography showed a hypokinetic left ventricular anterior wall. ICA was performed to rule out MI, showing normal coronary arteries (**A**) and a hypokinetic apex with mildly reduced LVEF of 45% (**B**, end-diastolic ventriculography; **C**, end-systolic ventriculography), suggestive of TTC. CMR performed 10 days later displayed normalization of LVEF and wall motion abnormalities (**D**, end-diastolic and **E**, end-systolic sequences on balanced SSFP cine sequences in 3-chambers view), and no sign of myocardial scar or edema on LGE and T1-mapping sequences (**F**, T1 inversion recovery LGE sequence in 3-chambers view) and was therefore not suggestive of TTC. Given the initial ECG and hypokinetic pattern suggestive of a transient reduced coronary flow in the LAD, coronary vasospasm was hypothesized. A second ICA with assessment of coronary microvascular function and coronary vasoreactivity testing was performed (**G**, ICA before pharmacological spasm provocation test). Assessment of coronary microvascular function confirmed normal coronary arteries (index of microcirculatory resistance = 10, N < 25; CFR = 4.5, N > 2). Following intracoronary infusion of 100 μg of acetylcholine, all 3 criteria for vasospastic angina were met, i.e., (i) a marked diffuse vasospasm, most pronounced in the proximal left anterior descending artery (**H**, red arrowhead), (ii) angina symptoms provoked by acetylcholine infusion, and (iii) ST-elevation on ECG. All these findings were completely reversed after administration of intracoronary nitroglycerin (**I**). Noteworthy, acetylcholine-induced coronary spasm can be induced at lower acetylcholine doses in women than in men, suggesting a higher sensitivity to vasospasms in the female population which could be related to the high prevalence of CMVD [225]. Upon discharge, a defibrillator was implanted and long-term treatment with calcium-channel blockers and long-acting nitrates was introduced along with advises to cease smoking, which constitutes an important risk factor for coronary vasospasm [226]. Given the fact that up to 80% of MINOCA patients are women, an epicardial origin to angina or, in this case, to cardiac arrest should not be dismissed before ruling out coronary vasospasm [227]. ICA-based criteria are well established to diagnose epicardial vasospastic angina and should be discussed after the acute phase when no clear explanation for an ACS in a woman can be evidenced. **Abbreviations**: *ACS*: acute coronary syndrome; *CFR*: coronary flow reserve; *CMR*: cardiac magnetic resonance; *CMVD*: coronary microvascular dysfunction; *ECG*: electrocardiogram; *ICA*: invasive coronary angiography; *LAD*: left anterior descending artery; *LGE*: late gadolinium enhancement; *LVEF*: left ventricular ejection fraction; *MI*: myocardial infarction; *MINOCA*: myocardial infarction with no obstructive coronary artery disease; *N*: normal; *SSFP*: steady-state free precession; *TTC*: Takotsubo cardiomyopathy

**Case 2:**
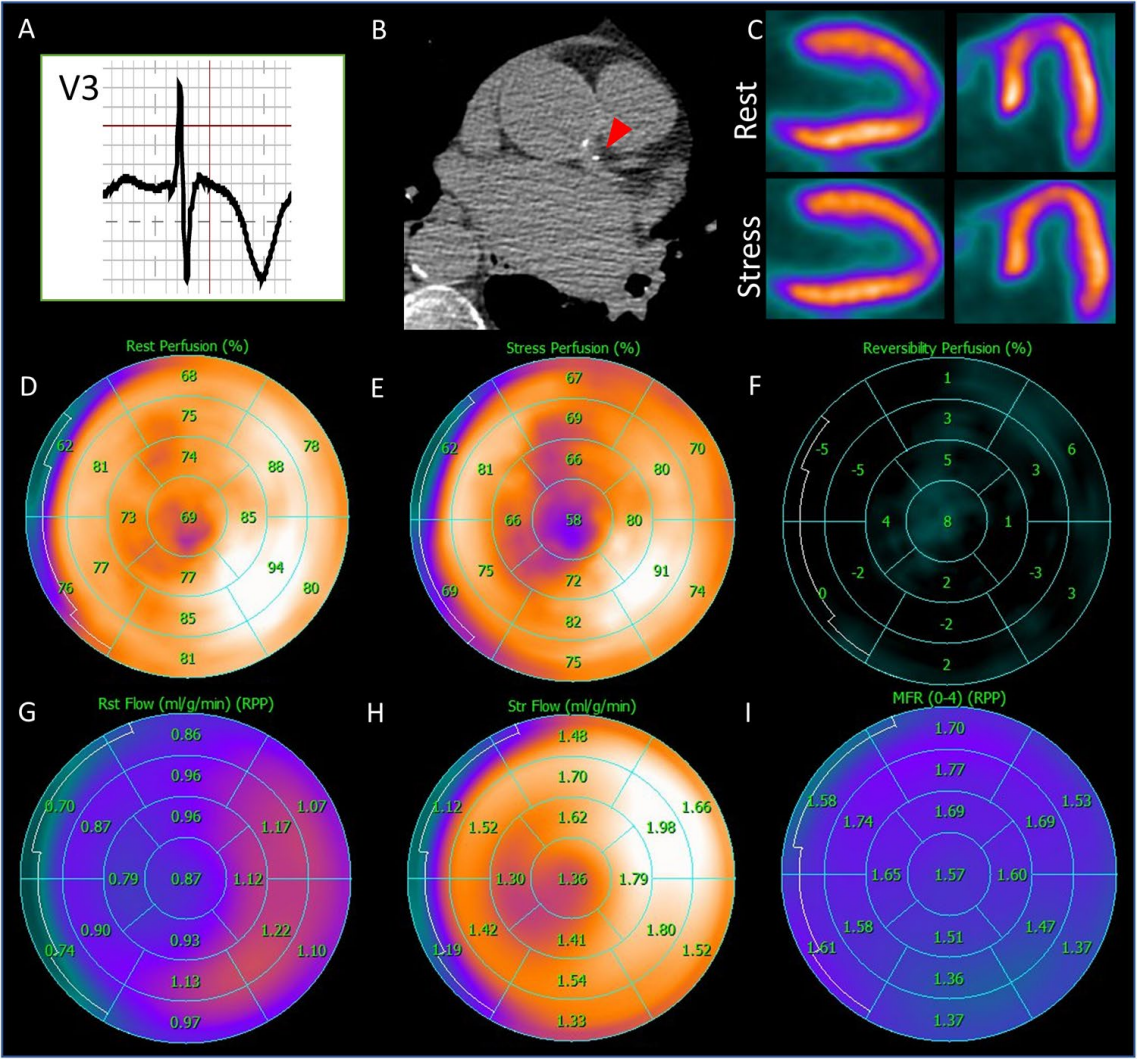
Chest pain in a 62-year-old woman. A 62-year-old woman with a history of hypertension and polycystic ovary syndrome presented with ongoing episodes of atypical chest pain (high abdominal discomfort) and shortness of breath on exertion. These symptoms had evolved for several years and had lately been increasing in frequency. A submaximal stress ECG test reproduced the symptoms accompanied by negative T-waves in the anterior leads (**A**). Subsequently, ^13^N-NH_3_-PET-MPI was performed because myocardial ischemia was suspected. CACS calculated from low-dose CT amounted to 5, due to minimal calcification of the left coronary artery (**B**, red arrowhead), indicating a low risk of cardiovascular mortality (score A1N1 of the CACS data and reporting system [228], with A1 indicating a mildly increased risk and N1 indicating the involvement of one single vessel). Analysis of relative perfusion showed homogenous ^13^N-NH_3_ uptake in all myocardial segments at rest and stress (**C**, horizontal and vertical long axes; **D** and **E**: polar maps), with no reversible or non-reversible perfusion defects (**F**, polar map), thereby making regional ischemia or scar in an epicardial territory unlikely. The CFR calculated from rest (**G**, polar map) and stress (**H**, polar map) MBF was diffusively reduced (< 2) in all myocardial segments (**I**, polar map). This finding was consistent with the diagnosis of CMVD-related microvascular angina. Subsequently, medical treatments of CMVD including betablockers and nitrates were introduced, alongside the control of CVRFs. Indeed, although to date no standardized treatment of CMVD exists, the current recommendations are to control factors that promote inflammation and thrombosis (such as statin, aspirin, and betablockers) as well as vasomotor dysfunction (such as nitrates) [229]. CMVD is present in about two-thirds of women with angina and non-obstructive CAD [27] and contributes to adverse cardiovascular outcomes in women [230]. Therefore, this diagnosis should always be evoked in women with persisting angina, even in the absence of significant epicardial coronary stenosis. Nuclear imaging techniques, especially PET-MPI, are of paramount importance to establish the diagnosis of CMVD. **Abbreviations**: ^13^*N-NH*_3_: ^13^*N-ammonia*; *CACS*: coronary artery calcium score;* CAD*: coronary artery disease; *CFR*: coronary flow reserve; *CMVD*: coronary microvascular dysfunction; *CT*: computed tomography; *CVRF*: cardiovascular risk factor; *ECG*: electrocardiogram; *MBF*: myocardial blood flow; *MPI*: myocardial perfusion imaging; *PET:* positron emission tomography

### Differential diagnosis of heart failure etiologies

#### Heart failure with preserved ejection fraction

The European Society of Cardiology (ESC) proposes a four-step algorithm to diagnose HFpEF [120]. First, women with dyspnea (especially on exertion) or orthopnea should undergo a pretest evaluation. This consists of screening for HFpEF risk factors such as obesity, metabolic syndrome/diabetes mellitus, physical inactivity, and arterial hypertension, recording ECG in search for atrial fibrillation (a highly predictive criteria for HFpEF) [138], and performing echocardiography or CMR showing LVEF ≥ 50%, non-dilated LV, concentric hypertrophy, and left atrial enlargement [120, 139] (Case 3). If the presentation is suggestive of HFpEF, an echocardiographic score is calculated based on a series of major and minor criteria which will not be detailed here [120]. In case the score does not allow ruling in or ruling out the diagnosis of HFpEF, a 3^rd^ step consists of considering diastolic dysfunction assessed by stress echocardiography, which alongside preserved LVEF is a cardinal feature of HFpEF [120, 139]. If the diagnosis still remains uncertain, invasive hemodynamic measurements using left and right heart catheterization are recommended with measurement of LV end-diastolic pressure and mean pulmonary capillary wedge pressure [120]. Once HFpEF is confirmed, the final step consists of an etiological workup. Given that CMVD is present in most patients with HFpEF [6], PET-MPI with calculation of CFR and MBF is recommended [120]. CMR detects most of the specific etiologies (ischemic cardiomyopathy, myocarditis, or restrictive cardiomyopathies), while CCTA can be most useful to evidence CAD [130]. In selected cases, bone scintigraphy and ^123^I-meta-iodobenzylguanidine (^123^I-MIBG) scintigraphy are useful to detect cardiac amyloidosis [140].

**Case 3:**
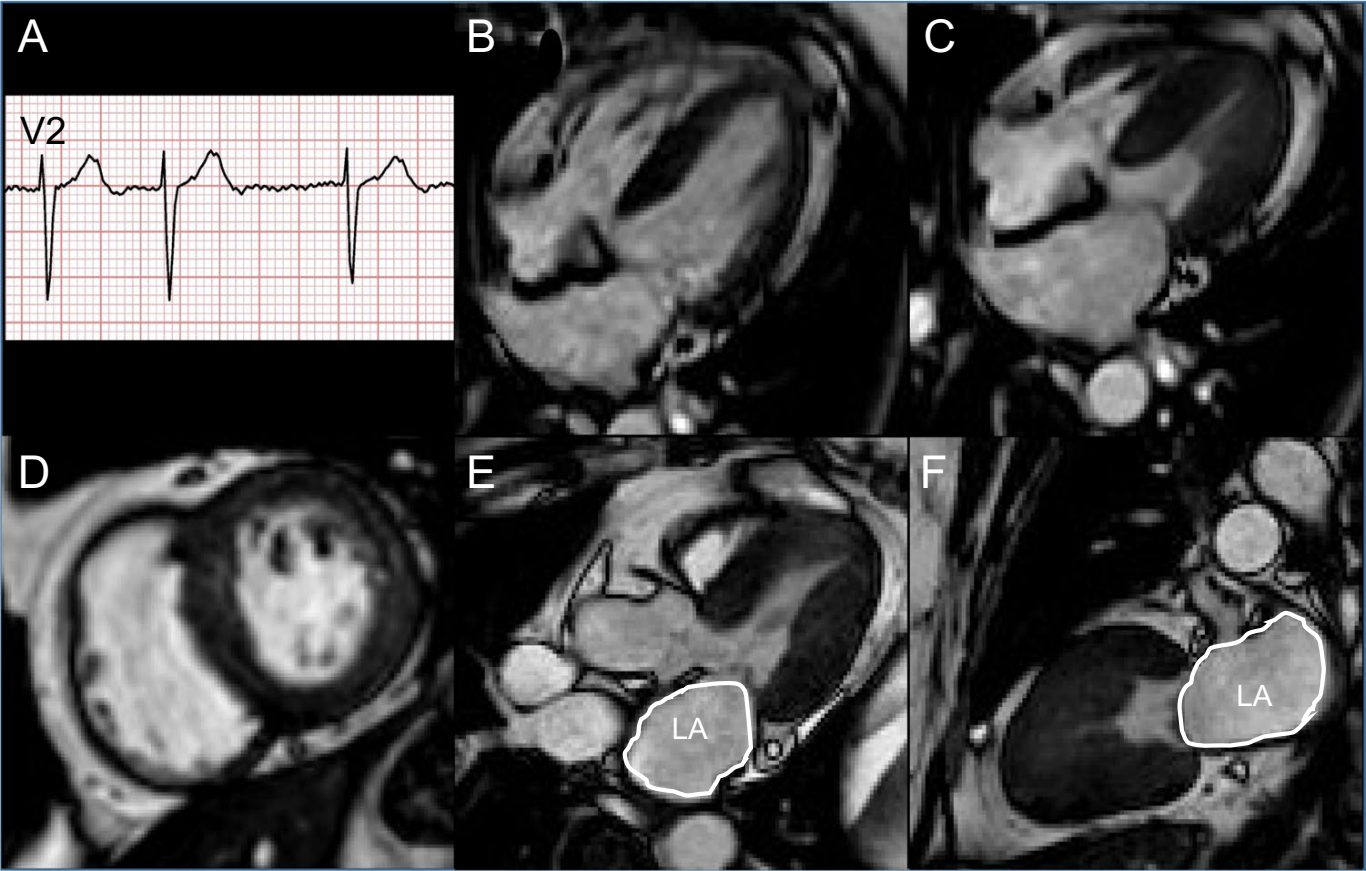
Chronic dyspnea in a 71-year-old woman. A 71-year-old woman with a history of WHO grade 3 obesity (body-mass-index 40.1 kg/m^2^) and type 2 diabetes was referred for worsening dyspnea (NYHA grade II-III). Clinically no other signs of congestion or fluid retention were observed. The ECG showed normofrequent atrial fibrillation (**A**). The NT-proBNP values were increased (1190 ng/L, N < 738 ng/L). Echocardiography displayed a preserved LVEF (60%) with concentric hypertrophic remodeling and type II diastolic dysfunction. CMR confirmed the preserved LVEF (55%) with a non-dilated (**B**, LV end-diastolic volume: 98 mL, N = 77–158 mL; **C**, LV end-systolic volume: 44 mL, N = 37–48 mL) hypertrophic LV (indexed LV mass = 58 g/m^2^, N < 55.9 g/m^2^), In addition, LGE imaging showed no scar (D) and a dilated LA was detected (23 cm^2^, N < 16.0 cm^2^; **E** and **F**, white contouring). Rest/stress perfusion showed no sign of ischemia or scar. Consequently, the diagnosis of HFpEF was established, and an appropriate therapy consisting of diuretics and angiotensin-converting enzymes inhibitor was initiated. Interestingly, new FDA-approved classes of medication, i.e., neprilysin inhibitor and empagliflozin [231], have recently been shown to reduce cardiovascular mortality and rate of re-hospitalization for HFpEF patients, with an effect of neprilysin inhibitor persisting for higher LVEF values in women than in men [232]. Comorbidities that are associated with inflammation, such as hypertension, diabetes, and obesity, play a central role in the development of HFpEF, particularly in women [233]. In addition, a systolic LV dysfunction is increasingly recognized as being a single aspect of HF, which can also result from diastolic dysfunction as reflected in this case. Therefore, HFpEF should always be kept in mind in women with dyspnea and comorbidities that favor inflammation. **Abbreviations**: *CMR*: cardiac magnetic resonance; *ECG*: electrocardiogram; *FDA*: Food and Drug Administration; *HF*: heart failure; *HFpEF*: heart failure with preserved ejection fraction; *LA*: left atrium; *LGE*: left gadolinium enhancement; *LV*: left ventricle; *LVEF*: left ventricular ejection fraction; *N*: normal; *NT-proBNP*: N-terminal brain natriuretic peptide; *WHO*: World Health Organization

#### Heart failure with reduced ejection fraction

In case of HFrEF, the lower rate of ischemic origin in women stresses the importance of CMR with LGE and T1/T2 mapping techniques to establish alternative etiologies, such as dilated cardiomyopathy (DCM), hypertrophic cardiomyopathy (HCM), valvular heart disease (VHD) [141–143], and arrhythmogenic right ventricular cardiomyopathy [144].

#### Takotsubo cardiomyopathy

Although invasive imaging establishes the diagnosis of TTC in most cases, by ruling out coronary obstruction and showing the hallmark wall motion abnormality [145], multimodality imaging plays a key role in the diagnosis of TTC [146]. Echocardiography is the first-line exam, displaying the characteristic LV wall motion abnormality generally extending beyond a coronary territory. ICA is often required owing to the MI-like presentation of TTC, showing normal coronary arteries. Alternatively, CCTA can be performed in stable patients, ruling out CAD with high confidence. CMR with LGE and mapping techniques is a particularly comprehensive tool, revealing the typical kinetic abnormalities, myocardial edema (with increased T2-weighted signal and T1 mapping values), excluding differential diagnosis (particularly MINOCA and myocarditis), and detecting complications such as LV thrombi. Often overlooked, nuclear imaging tools can also establish the diagnosis, particularly in the subacute phase when wall motion has normalized thereby misleading other imaging tools (Case 4). In the subacute phase, nuclear MPI shows preserved perfusion, while ^123^I-MIBG and ^18^F-fluorodeoxyglucose (^18^F-FDG) uptakes remain reduced despite normalization of ventricular kinetics [147]. Interestingly, 6-Fluoro-[18F]-l-3,4-dihydroxyphenylalanine (^18^F-DOPA) PET-based studies have documented an age-dependent increase in ^18^F-DOPA uptake in the LV apex of women, which was not present in men, indicating an enhanced sympathetic activity which could account for the higher susceptibility of postmenopausal women to TTC [18].

**Case 4:**
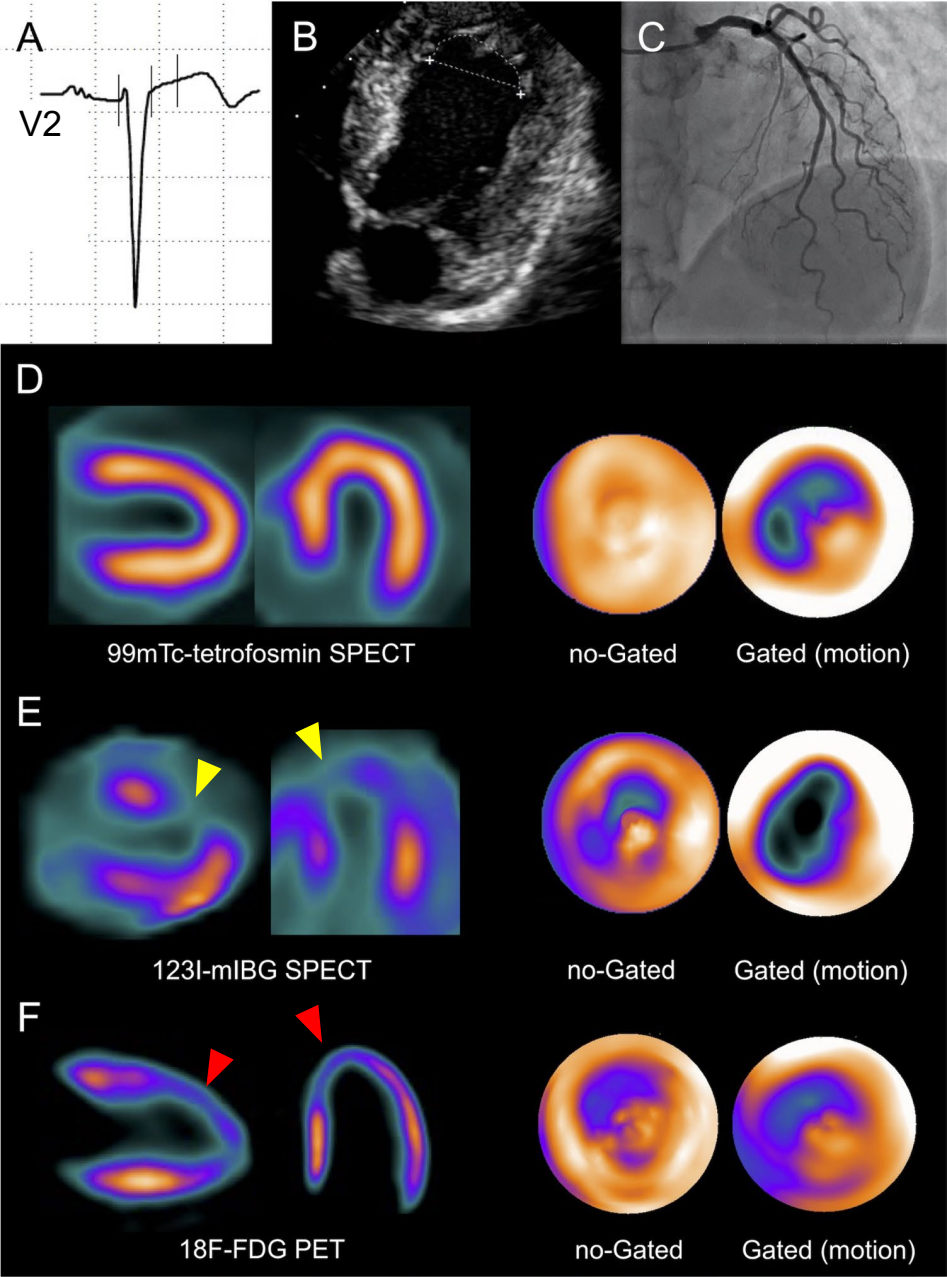
Sixty-year-old woman for routine checkup. A 60-year-old woman with severe hypercholesterolemia presented to the cardiology outpatient clinic for a routine evaluation. The resting ECG (**A**, V3 lead) showed a loss of R-wave progression and biphasic T-waves in the anterior precordial and lateral leads, respectively. Echocardiography (**B**) revealed isolated apical akinesia with preserved LVEF. Subsequent ICA was normal (**C**). ECG-gated ^99m^Tc-MPI-SPECT performed 7 days later showed normal LV stress/rest perfusion, but reduced end-systolic thickening and antero-apical hypokinesia (**D**, horizontal and vertical long axes, polar maps). TTC was hypothesized and, 2 weeks after the perfusion scan, the patient underwent ECG-gated ^123^I-MIBG SPECT (**E**) and ^18^F-FDG PET/CT (**F**), showing a concordant alteration of adrenergic innervation (**E**, yellow arrowheads) and glucose metabolism (**F**, red arrowheads) in apical and anterior LV wall. A careful patient questioning revealed significant stress exposure at work triggering the (clinically silent) TTC. The resolution of the workplace conflict coincided with the recovery of LV apical kinetics documented by echocardiography 3 months later. TTC is often caused by severe emotional stress. If segmental LV wall motion disorders are present, an adrenergic cause must be considered. In this context, nuclear medicine provides a specific imaging pattern, useful to reach the diagnosis. **Abbreviations**: ^18^*F-FDG: *Fluor-18- radiolabeled fluorodeoxyglucose; ^99m^*Tc*: ^99m^Technetium; ^123^*I-MIBG*: ^123^I-meta-iodobenzylguanidine; *CT*: computed tomography; *ECG*: electrocardiogram; *ICA*: invasive coronary angiography; *LV*: left ventricle; *LVEF*: left ventricular ejection fraction; *MPI*: myocardial perfusion imaging; *PET*: positron emission tomography; *SPECT*: single-photon emission computed tomography; *TTC*: Takotsubo cardiomyopathy

### Pregnancy

To date, heart diseases affect up to 4% of all pregnant women and 16% of pregnant women with preexisting cardiac conditions [148], rendering CVD the leading cause of maternal morbidity [149]. The latter represents one-third of all pregnancy-related maternal deaths [150]. This alarming situation has been in part attributed to the growing number of pregnancies in women aged > 35 years and to the prolonged survival of women with congenital heart diseases reaching childbearing age [149]. Additionally, pregnancy complications such as hypertensive disorders, preterm delivery, and gestational diabetes occur more often in pregnant women > 40 years [151] and increase subsequent cardiovascular risk [152]. Consequently, evidence-based recommendations for pregnant women at increased cardiovascular risk have been established, in which cardiac imaging plays a central role [153, 154].

Pregnancy-related heart disease can be caused by a preexisting maternal condition that is either first diagnosed or has worsened during pregnancy [148, 154] or by a condition newly acquired during pregnancy [155] such as PPCM (Case 5) or peripartum SCAD (Case 6).

**Case 5:**
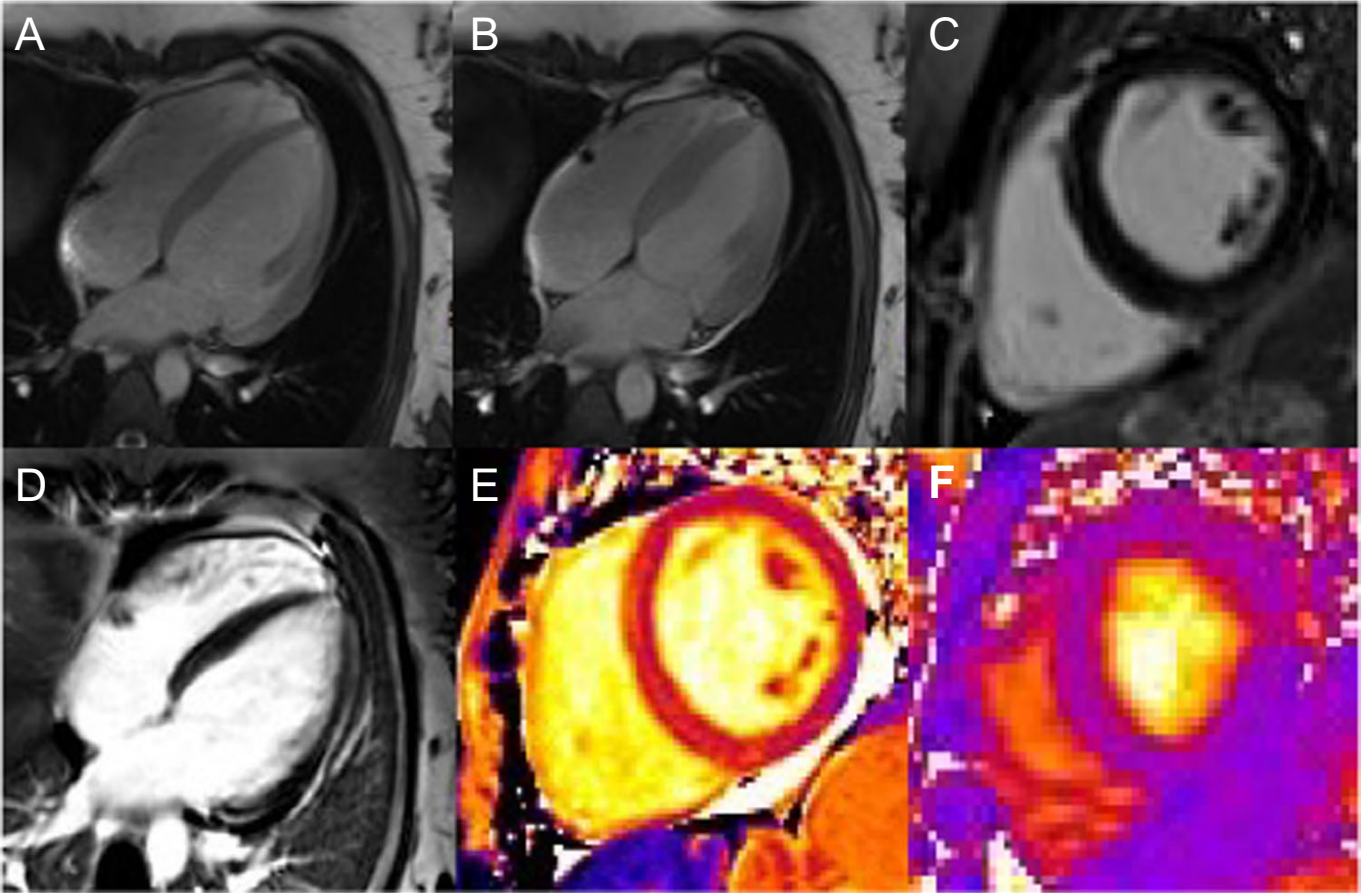
Non-sustained ventricular tachyarrhythmia and dyspnea in a 31-year-old woman. A 31-year-old primiparous woman with a history of polycystic ovary syndrome and obesity (body mass index 33 kg/m^2^) and no family history of CVD was referred to hospital for a planned caesarian section. Her pregnancy has been complicated by hypertension and gestational diabetes. The aftermath of delivery was marked by episodes of non-sustained ventricular tachyarrhythmia and dyspnea. Troponin and NT-proBNP were within normal range. An echocardiography revealed a mildly reduced LVEF (40%) without regional wall motion abnormalities and relevant valvular abnormality, as well as normal right-sided cardiac cavities. CMR was performed confirming a mildly reduced LVEF of 42% (panels **A** and **B**) with no regional component, particularly no pattern suggestive of TTC. LV and RV were non-dilated (respectively, LV end-diastolic volume 85 mL/m^2^, N: 66–101 mL/m^2^; and RV-end-diastolic volume 80 mL/m^2^, N: 65–111 mL/m^2^), hence excluding preexisting cardiomyopathies and DCM. No myocardial LGE was detected (**C**), excluding necrosis, and pharmacological stress revealed no ischemia (**D**). T1 (**E**) and T2 (**F**) mapping of the LV myocardium were normal, 1247 ms (N: 1222 ± 42 ms) and 39 ms (N: 38 ± 2.3 ms), respectively, thus excluding scar and edema/myocarditis. Based on the timing of the disease (early postpartum) and other cardiomyopathies being excluded, the diagnosis of PPCM was made. Dyspnea during or in the direct aftermath of pregnancy is a common situation, which can result either from hemodynamic adaptations to pregnancy or from complications. The differential diagnosis in this setting includes pulmonary embolism and PPCM. Key to the diagnosis of PPCM is the timing of the disease and the absence of previous cardiomyopathies. Advanced noninvasive imaging is helpful in this setting, to establish LV systolic dysfunction and to rule out concurrent cardiomyopathies. **Abbreviations**: *CVD*: cardiovascular disease; *CMR*: cardiac magnetic resonance; *DCM*: dilated cardiomyopathy; *LGE*: late gadolinium enhancement; *LV*: left ventricle; *LVEF*: left ventricular ejection fraction; *N*: normal; *NT-proBNP*: N-terminal brain natriuretic peptide; *PPCM*: peripartum cardiomyopathy; *RV*: right ventricle; *TTC*: Takotsubo cardiomyopathy

**Case 6:**
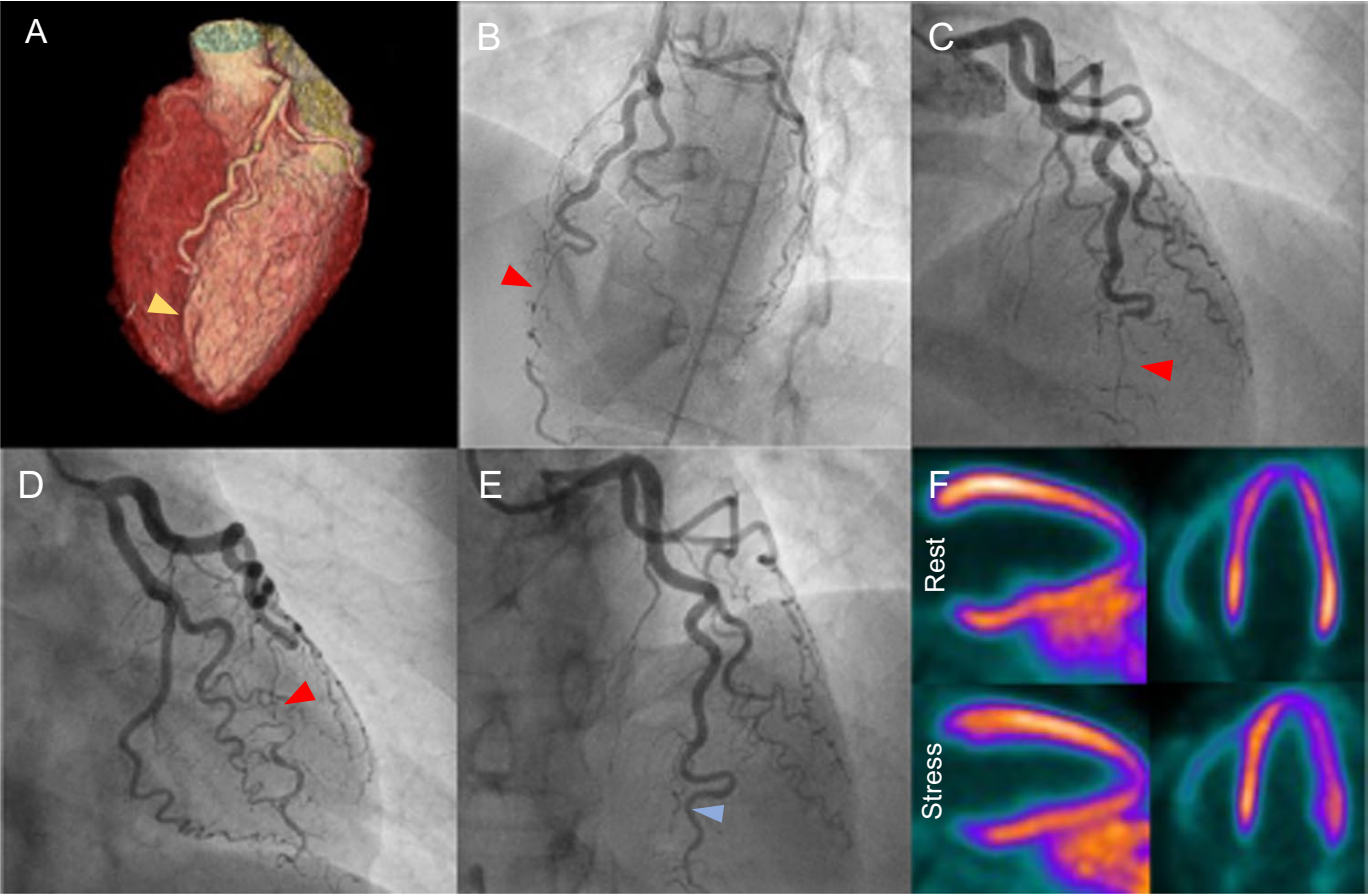
Intermittent retrosternal pain in a 30-year-old woman, 6 weeks postpartum. A 30-year-old female patient, 6 weeks postpartum after an uncomplicated pregnancy with no prior medical history or CVRFs, presented to the emergency department for intermittent retrosternal pain which had evolved over 2 weeks. Admission workup revealed a mildly elevated troponin (0.64 µg/L, N < 0.10 µg/L) while the ECG showed no abnormality suggestive of myocardial ischemia. The patient, being at low clinical likelihood of CAD, was referred for CCTA using a triple rule-out setting. The diagnoses of pulmonary embolism and aortic dissection were excluded. Coronary analysis showed no evidence of calcification or stenosis but revealed a complete occlusion of the distal segment of the left anterior descending artery (**A**, yellow arrowhead, volume rendering reconstruction of the heart and the coronary tree), compatible with either a SCAD or a thrombotic occlusion. The patient was referred for urgent ICA which confirmed the diagnosis of SCAD (**B**-**D**, red arrowheads), without signs of regional or diffuse hypokinesia in the ventriculography. A conservative strategy was initiated, combining anticoagulation with dual antiplatelet therapy. After the acute episode, a whole-body angio-magnetic resonance imaging was performed that found no evidence of fibromuscular dysplasia. Genetic analysis ruled out genetic disorders. ICA was repeated after 6 weeks, confirming spontaneous partial revascularization of the dissected artery with persistence of a 50% residual stenosis with TIMI III post-stenotic flow (**E**, blue arrowhead), treated conservatively by continuation of dual antiplatelet therapy. ^13^N-NH_3_-PET-MPI was also realized, showing no signs of scar or ischemia (**F**, horizontal and vertical long axes, at rest and stress). Although rare, SCAD is a classical cause of ACS in the late stages of pregnancy and in the early postpartum period. Therefore, SCAD should be suspected in young women without cardiovascular risk factors presenting with ACS or cardiac arrest. While CCTA is preferred in hemodynamically stable patients, ICA is often required to establish the diagnosis. Noninvasive imaging can also be useful to screen for predisposing vascular diseases and to exclude differential diagnosis of chest pain in young women, such as pulmonary embolism. **Abbreviations**: ^13^*N-NH*_3_: asnitrogen-13-radiolabeled ammonia; *ACS*: acute coronary syndrome; *CAD*: coronary artery disease; *CCTA*: coronary computed tomography angiography; *CVRF*: cardiovascular risk factor; *ECG*: electrocardiogram; *ICA*: invasive coronary angiography; *MPI*: myocardial perfusion imaging; *PET;* positron emission tomography; *SCAD*: spontaneous coronary artery dissection; *TIMI*: thrombolysis in myocardial infarction score

#### Challenges of cardiac imaging in pregnant women

Two main challenges must be addressed when imaging pregnant women: (1) distinguishing pathological conditions from the physiological changes occurring during pregnancy; and (2) finding the optimal trade-off between radiation exposure and image quality [156].

During pregnancy, physiological adaptive cardiovascular changes occur [149]. These changes consist mainly of an increase in LV end-diastolic volume, LV mass, and cardiac output [157]. In addition, one out of four pregnant women shows an increase in LV trabeculation, which can mimic LV non-compaction cardiomyopathy [158]. Pregnancy is also associated with tachycardia [157], which can represent a technical limitation for ECG gating.

Radiation exposure during pregnancy increases the risk of breast cancer in the mother [159] and may induce miscarriage, malformation, or fetal cancer [160]. Consequently, pregnant women should preferentially undergo non-ionizing imaging, i.e., echocardiography or non-contrast-enhanced CMR [161]. It is nevertheless recommended to avoid magnetic resonance imaging > 1.5 Tesla due to concerns related to heating effect, especially during the 1^st^ trimester [69]. Additionally, fetal exposure to gadolinium at whatever stage of pregnancy increases the risk of stillbirth or neonatal death and should be limited to the only cases when the expected benefits clearly outweigh the risks [162].

If necessary, and in agreement with a fully informed patient, ionizing techniques should not be withheld from pregnant patients, together with or in replacement of non-ionizing techniques [162]. Indeed, although no threshold exists below which there would be no risk for the fetus (stochastic effect), the risk associated with fetal radiation < 100 milliGray is considered negligible, which is beyond the usual radiation doses of diagnostic imaging [163, 164]. The *as low as reasonably achievable* (ALARA) dose should always be the guiding principle (ideally < 50 milliGray [165]) while preserving image quality. This can be achieved by preferentially resorting to non-ionizing imaging, by optimizing CT parameters (preferential use of low voltage and of high pitch, careful selection of tube current, use of novel reconstruction algorithms, avoiding multiple phases imaging), and for nuclear imaging by opting for radiotracers with shorter half-life (^99m^Tc rather than ^201^Tl [166]) and reducing the injected radiotracer activity [66, 167]. In case scintigraphy is performed, breastfeeding needs to be interrupted for > 12 h after the study due to the excretion of the radiotracer in the maternal milk [136].

Positioning during image acquisition is also important. Indeed, in the supine position, the gravid uterus can compress the inferior vena cava which may affect cardiac output; to prevent this, pregnant women should undergo imaging in the left lateral tilt position [168].

### Specific cardiac diseases in pregnancy

#### Peripartum cardiomyopathy

The incidence of PPCM is 1 per 1000–4000 live births in Western countries, but its incidence varies depending on predisposing factors including age > 30 years, black ethnicity, preeclampsia, hypertension, and multiparity [169]. Several pathophysiological hypotheses have been proposed to account for the development of PPCM (vascular, hormonal, and genetic) [170], but the exact underlying causes remain debated.

PPCM is defined as symptomatic LV systolic dysfunction with LVEF < 45%, developing either in the last month of pregnancy or in the 5 months following its end in women with no previously documented cardiac disease [171]. A simplified definition describes PPCM as an idiopathic HF developing towards the end of pregnancy or in the months following delivery [172]. This definition limits the risk of PPCM being under-diagnosed by too stringent timeframe cutoffs. Noteworthy, LVEF can be preserved or mildly impaired in early stages of PPCM [173]. Given the broadness of the diagnostic criteria, PPCM is in practice an exclusion diagnosis, retained after ruling out other cardiomyopathies and preexisting heart diseases, especially DCM that shares common clinical and genetic features with PPCM [174, 175]. The time of onset can help distinguishing both entities. DCM develops preferentially during late pregnancy, whereas PPCM usually occurs in the early weeks after delivery [176]. DCM must also be considered in case of familial history or preexisting LV dysfunction [177].

PPCM is usually reversible within 6 months after termination of pregnancy [178]. Factors associated with a lower rate of 12-month event-free survival or reduced LV functional recovery are LVEF < 30% at diagnosis, LV end-diastolic diameter > 60 mm, an involvement of the right ventricle (RV), and evidence of myocardial edema on CMR [179–181].

The frontline imaging exam is echocardiography [181]. CMR also accurately measures cardiac volumes, and T2 imaging characterizes myocardial edema in postpartum women [182]. Moreover, CMR excludes differential diagnosis, such as myocarditis, TTC, and DCM, and reveals stigmas of previous cardiomyopathies [141, 154, 171, 181]. CMR can also identify LV thrombi, a classical complication of PPCM [183]. LGE can evidence myocardial scarring (in postpartum women when gadolinium can safely be used), which is associated with worse outcomes [184]. CMR screens for predictors of impaired prognosis such as RV involvement [180], and mid-myocardial local LGE [185] although the prognostic value of LGE in PPCM is debated [179, 181]. CMR can further be used to monitor functional recovery, thereby guiding the therapeutic strategy [154, 177] which consists of cautious use of conventional HF medications, taking into account the risk to the child during pregnancy or related to breastfeeding, as well as of bromocriptine [186].

Ionizing techniques are usually not needed to diagnose PPCM. However, they can be useful to exclude other causes of dyspnea and HF during pregnancy, mainly pulmonary embolism and CAD [181].

#### Spontaneous coronary artery dissection

SCAD is an important cause of MI, especially in young and middle-aged women, and during pregnancy [187]. The reasons behind this sex dimorphism are not clear and could be linked to sex hormones as well as distinct susceptibility genes [187]. Although no sex chromosome-related gene has been identified, the overrepresentation of women in SCAD raises the question whether genes with estrogen response elements are implicated in the pathophysiology of this condition [187]. In pregnant women, the risk of SCAD is particularly high in the first week postpartum [188]. While the diagnosis is usually made by ICA, optical coherence tomography (OCT) and intravascular ultrasound are helpful in cases of uncertainty or to guide revascularization [187]. CCTA can show abrupt luminal interruption [187] and should be preferred in hemodynamically stable women, owing to the risk of aggravating the dissection by contrast injection in the coronary ostium during ICA [187]. CCTA typical findings include an intimo-medial flap, lumen narrowing, or even occlusion, caused by thrombosis or intramural hematoma [189].

### Cancer

CVD is the leading cause of morbidity and mortality of cancer survivors, affecting one out of three patients [190]. Of note, female survivors of breast and cervix cancer face a 20–30% higher risk of cardiac deaths than the general population [190]. Detecting patients at risk for cardiotoxicity allows early initiation of cardioprotective therapies or avoidance of highly cardiotoxic drugs, thereby alleviating the burden of CVD in cancer patients [191]. The links between the heart and neoplastic diseases can be direct (metastasis and primary tumors) or indirect, with the latter consisting mainly of cardiotoxicity related either to therapy or to circulating factors (such as amyloid deposits in amyloidosis or serotonin in carcinoid tumors) [192].

Female sex is one of the most important risk factors of cancer treatment-related cardiotoxicity [193]. Indeed, cancer drugs that are commonly associated with cardiotoxicity include anthracyclines, alkylating agents, platinum compounds, monoclonal antibodies (notably trastuzumab), and antibody–drug conjugates, all of which are cornerstone treatments of female cancers [194]. In addition, hormonal treatments such as aromatase inhibitors specifically used in female hormone-dependent cancers might favor CVD [195]. Breast cancer is also associated with an increased risk of radiotherapy-induced cardiotoxicity due to its proximity to the heart, a risk that is potentiated by the concomitant use of anthracyclines [196].

### Different types of cancer-related cardiovascular complications

#### Cancer therapy-related cardiac dysfunction

Cancer therapy-related cardiac dysfunction (CTRCD) is the most common complication of cancer therapy (Case 7). CTRCD is defined as a drop in systolic LV function below the lower limit of normal (< 50% for the ESC, < 53% for the European Association of Cardiovascular Imaging) in the context of cancer treatment administration along with a > 10% decline from baseline value. Confirmation by repeating the study 2–3 weeks after initial diagnosis must be obtained [197]. An additional mandatory criterion is a reduction in global longitudinal strain (GLS) > 15% from baseline [197]. Guidelines do not distinguish male and female LVEF thresholds for the diagnosis of CTRCD. However, active breast cancer in chemotherapy-naïve patients is associated with increased strain amplitude and systolic strain rate [198]. This, in conjunction with the higher values of LVEF in postmenopausal women, highlights the need for specific cut-off values of LVEF and GLS in women to avoid missing early CTRCD signs.

**Case 7:**
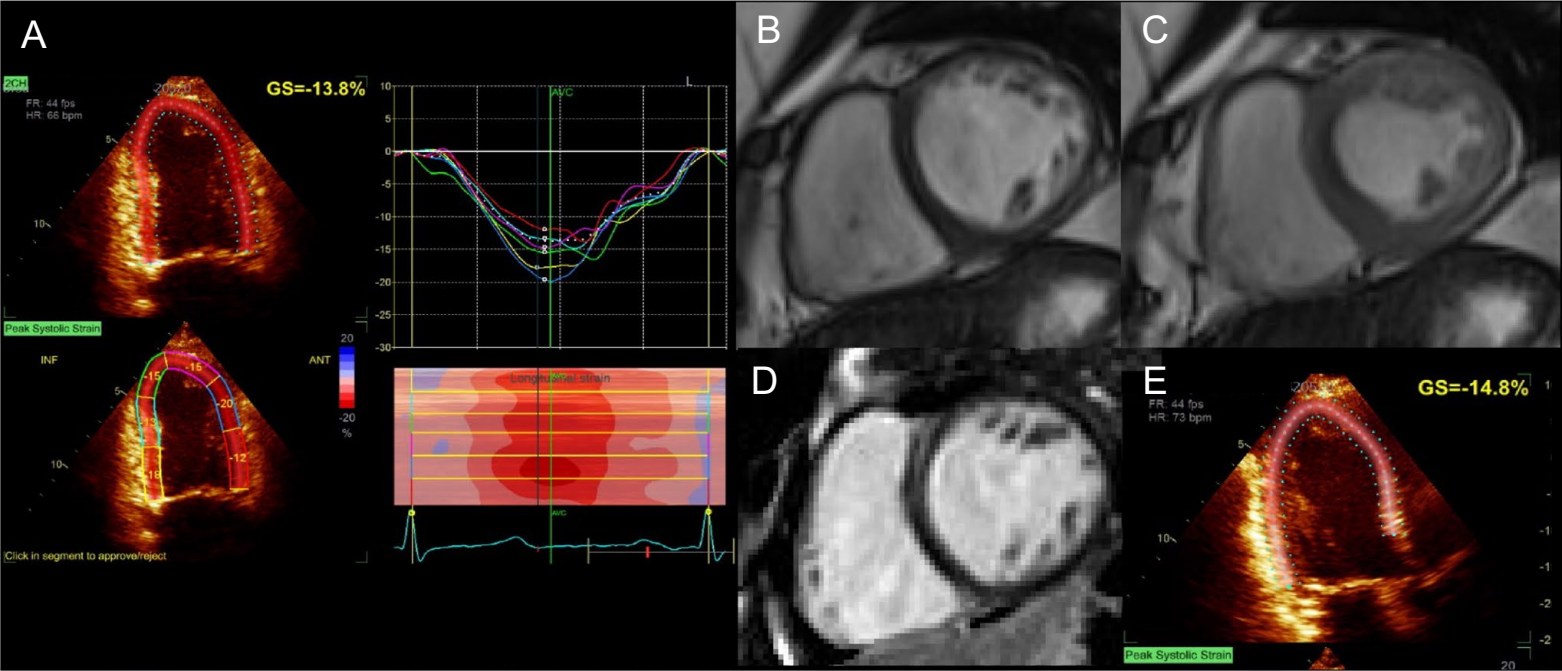
Acute onset of dyspnea in a 47-year-old woman with breast cancer. A 74-year-old woman without CVRFs presented with a human epidermal growth factor receptor 2 (HER2)-positive, hormone receptor (HR)-negative, locally advanced left-sided breast cancer with ipsilateral axillary lymph node extension (pT2N1M0, stage IIB). Given her age and the absence of indication for radiotherapy, she underwent surgical treatment by mastectomy and axillary lymph node dissection. Adjuvant chemotherapy was planned, consisting of 4 cycles doxorubicin (60 mg/m^2^) plus cyclophosphamide (500 mg/m^2^) and 12 cycles of weekly paclitaxel (75 mg/m^2^) plus trastuzumab (2 mg/kg) followed by 1 year of trastuzumab maintenance (3-weekly, 6 mg/kg). Pre-treatment echocardiography showed a normal LVEF (65%) and a normal GLS (18%). Following the third cycle [cumulative dose of anthracycline 180 mg/m^2^ (recommended maximum cumulative dose 400 mg/m^2^)], the patient presented with an acute onset of dyspnea requiring oxygen therapy. NT-proBNP and troponin I were increased (1026 ng/mL, ULN 738 ng/mL and 0.26 μg/L, ULN 0.10 μg/L, respectively). Echocardiography showed decreased LVEF (45%) and GLS (13.8%, N > 15%). **A** Doxorubicin-induced CTRCD was suspected, and a CMR was performed confirming reduced LVEF (42%). **B** and **C** Balanced SSFP short-axis view of mid-LV. LGE sequences showed no sign of scar (**D**). Chemotherapy was withheld and HF treatments consisting of diuretic, betablockers, and angiotensin-converting enzyme inhibitor were initiated, and control echocardiography showed an increase of LVEF but persistence of mildly decreased GLS (**E**), indicating persistent subclinical cardiotoxicity. Any further exposure to doxorubicin was omitted. Continuation of the treatment with paclitaxel and trastuzumab was planned to be initiated when LVEF would recover to approximately 50% under regular echocardiographic and laboratory monitoring. With breast cancer becoming the most prevalent cancer worldwide in 2020 [234], a shift of the overall burden of cancer treatment-related cardiac complications towards women can be expected. Increasing awareness about the intertwining of cardiac diseases and cancer is therefore paramount to cardiac imagers. The detection of subclinical cardiotoxicity before the onset of heart failure using advanced imaging criteria, such as GLS, or obtaining cardiovascular information from oncological imaging exams [213] could help to reduce the complications of cancer treatments and should therefore be part of the routine monitoring of patients undergoing potentially cardiotoxic therapies. **Abbreviations**: *CMR*: cardiac magnetic resonance;* CTRCD*: cancer treatment-related cardiac disease; *CVRF*: cardiovascular risk factor; *GLS*: global longitudinal strain; *HF*: heart failure; *LGE*: late gadolinium enhancement; *LV*: left ventricle; *LVEF*: left ventricular ejection fraction; *NT-proBNP*: N-terminal brain natriuretic peptide; *SSFP*: steady-state free precession; *ULN*: upper limit of normal

While endomyocardial biopsy is the gold standard to diagnose CTRCD, noninvasive imaging is the most common diagnostic tool. Echocardiography is the frontline exam to measure LVEF and GLS [199]. Nevertheless, echocardiography can be limited in women because of a smaller acoustic window related to breast interference, especially in case of implants, a common situation after breast cancer [200]. Breast implants and reconstructive surgery also limit the quality of apical-view-based GLS measurement [201]. CMR provides a more accurate and reproducible measurement of ventricular volumes as well as of GLS than echocardiography [202] and is not affected by breast artifacts [87]. Additionally, ECV and native T1 mapping can detect signs of myocardial fibrosis in women with breast cancer exposed to anthracycline [203]. Interestingly, CMR-derived LVEF and strain are predictive of subsequent CTRCD in early stage HER2 + breast cancer receiving sequential anthracycline/trastuzumab [204]. A baseline percentage of normal myocardium ≥ 80% on CMR, defined as the percentage of LV myocardium with strain less than or equal to − 17%, helps identifying women with breast cancer at risk of CTRCD [205]. In those who do develop cardiotoxicity, normal myocardium ≥ 50% is predictive of recovery [205]. Although promising for the detection of subclinical cardiotoxicity, mapping techniques and ECV values tend to overlap between patients with and without CTRCD and need further evaluation [206]. Noteworthy, cancer itself and particularly breast cancer are associated with structural cardiac changes, such as smaller chamber sizes although without significantly affecting overall LVEF [198]. Additionally, breast cancer radiotherapy damages the coronary microvascular endothelium, hence promoting CMVD [207], a situation where high-resolution perfusion CMR could prove useful [74].

Alternatively, ERNA can be used to measure and monitor LVEF [208]. However, it should be noted that, in breast cancer patients receiving trastuzumab, ERNA-derived LVEF values are lower than those of CMR [209]. Nevertheless, the use of scintigraphy in women suffers limitations, related to breast tissue/implant attenuation, to the cumulative radiation exposure induced by serial follow-up, and to the fact that GLS cannot be derived from ERNA.

#### Cancer therapy-related ischemic heart disease

Anticancer treatment can lead to other myocardial diseases including CAD. The risk of CAD is particularly increased in women with left breast cancer undergoing radiotherapy, which can induce fibrous stenosis of the left anterior descending artery, and accelerates progression of existing plaques [210]. Similarly, atherosclerosis progression is accelerated in patients receiving alkylating-like agents, fluoropyrimidines, and platinum compounds (increasing the risk of arterial thrombosis and vasospasm) [211]. Interestingly, CT scan used to plan radiotherapy for breast cancer also enables measuring CACS, an independent predictor of ischemic heart disease [212]. Furthermore, a recent study has shown that ^18^F-FDG myocardial uptake pattern is predictive of altered myocardial perfusion. Using this information obtained from oncologic imaging exams might therefore be helpful in identifying patients at risk for future cardiovascular complications of anticancer therapy at an early stage [213].

#### Valvular heart disease

VHD in cancer patients consist mainly of treatment-induced fibrosis/calcification leading to stenosis/regurgitation [214], and of cardiac masses obstructing the valves [215]. The risk of iatrogenic VHD is particularly marked in patients undergoing radiotherapy [215], and those receiving anthracycline [215, 216], hence in women treated for breast cancer. Echocardiography is the first-line modality to assess valve function [217]. CMR is the preferred alternative, particularly for regurgitant VHD [218]. CT is increasingly used in VHD, especially for the quantification of calcification and measurement of valve orifice area in aortic stenosis [219].

## Future directions

Lately, artificial intelligence (AI)-based machine learning (ML) techniques have emerged as a promising tool in the field of imaging, opening up unprecedented possibilities in cardiovascular imaging [220]. In a recent study, Baumann et al. compared the diagnostic performance of ML-based FFR-CT to detect lesion-specific ischemia between men and women [221]. ML-based FFR-CT correlated equally well with invasive FFR for both women and men. However, compared to CCTA, the diagnostic performances for the detection of ischemia were significantly better in men only. A potential explanation could be that the smaller diameter of coronary arteries in women might affect FFR-CT calculation.

AI could also be beneficial in patients with HFpEF. In a subgroup of predominantly female HFpEF patients with few risk factors for the condition, ML techniques identified an iron overload state, thus providing the means to assess pathophysiological pathways for HFpEF in women [222].

The last decade has witnessed the development of PET probes targeting sex hormone receptors in women with gynecological cancers, such as ^18^F-fluoro-17β-estradiol and ^18^F-fluoro-5α-dihydrotestosterone [223, 224]. Given the impact of sex hormones on cardiovascular health, the use of such probes potentially constitutes an interesting research tool in the field of gender-specific medicine.

## Conclusion

This review highlights sex-specific considerations that are critical for selecting the most appropriate cardiac imaging modality—with particular focus on challenges and opportunities of contemporary CVD management in women. Indeed, awareness about female attributes in cardiac imaging, considering technical implications and female-specific conditions, might help alleviate the burden of CVD in this subpopulation. Consequently, there is an urgent need for imaging guidelines that are tailored to women and men. While efforts have been made in this direction, substantial knowledge gaps still exist. Future imaging studies and recommendations require the integration of sex as an algorithm-modifying variable. In the era of precision medicine, accounting for sex disparities seems crucial to provide the best possible cardiovascular care to women and men.

## Abbreviations

^13^N-NH_3_: Nitrogen-13-radiolabeled ammonia
^15^O-H_2_O: Oxygen-15-radiolabeled water
^18^F-DOPA: 6-Fluoro-[18F]-l-3,4-dihydroxyphenylalanine
^18^F-FDG: Fluor-18-radiolabeled fluorodeoxyglucose
^18^F-Na: Fluor-18-sodium fluoride
^82^Rb: Rubidium-82
^123^I-MIBG: Iodine-123-meta-iodobenzylguanidine
^99m^Tc: Technetium-99m
ACS: Acute coronary syndrome
AI: Artificial intelligence
ALARA: As low as reasonably achievable
ARVC: Arrhythmogenic right ventricle cardiomyopathy
CACS: Coronary artery calcium score
CAD: Coronary artery disease
CCS: Chronic coronary syndrome
CCTA: Coronary computed tomography angiography
CFR: Coronary flow reserve
CMR: Cardiac magnetic resonance
CMVD: Coronary microvascular dysfunction
CT: Computed tomography
CTRCD: Cancer treatment-related cardiac dysfunction
CVD: Cardiovascular diseases
CVRF: Cardiovascular risk factor
DCM: Dilated cardiomyopathy
ECV: Extracellular volume
ECG: Electrocardiogram
ERNA: Equilibrium radionuclide angiocardiography
ESC: European Society of Cardiology
FFR: Fractional flow reserve
GLS: Global longitudinal strain
HCM: Hypertrophic cardiomyopathy
HF: Heart failure
HFmrEF: Heart failure with mildly reduced ejection fraction
HFpEF: Heart failure with preserved ejection fraction
HFrEF: Heart failure with reduced ejection fraction
ICA: Invasive coronary angiography
ICI: Immune checkpoint inhibitors
INOCA: Ischemia with no obstructive coronary arteries
IVUS: Intravascular ultrasound
LA: Left atrium
LGE: Late gadolinium enhancement
LV: Left ventricle
LVEDP: Left ventricular end-diastolic pressure
LVEF: Left ventricular ejection fraction
LVEDP: Left ventricular end-diastolic pressure
MACE: Major adverse cardiovascular events
MBF: Myocardial blood flow
MI: Myocardial infarction
MINOCA: Myocardial infarction with no obstructive coronary arteries
ML: Machine learning
mPCWP: Mean pulmonary capillary wedge pressure
MPI: Myocardial perfusion imaging
OCT: Optical coherence tomography
PET: Positron emission tomography
PPCM: Peripartum cardiomyopathy
RV: Right ventricle
SCAD: Spontaneous coronary artery dissection
SPECT: Single-photon emission computed tomography
SSFP: Steady state free precession
TTC: Takotsubo cardiomyopathy
TTE: Transesophageal echocardiography
